# A Rational Design of α-Helix-Shaped Peptides Employing the Hydrogen-Bond Surrogate Approach: A Modulation Strategy for Ras-RasGRF1 Interaction in Neuropsychiatric Disorders

**DOI:** 10.3390/ph14111099

**Published:** 2021-10-28

**Authors:** Maria Rita Gulotta, Riccardo Brambilla, Ugo Perricone, Andrea Brancale

**Affiliations:** 1 Molecular Informatics Unit, Fondazione Ri.MED, via Filippo Marini 14, 90128 Palermo, Italy; uperricone@fondazionerimed.com; 2 Neuroscience and Mental Health Research Institute (NMHRI) and Neuroscience Division, School of Biosciences, Cardiff University, Haydn Ellis Building, Maindy Road, Cardiff CF24 4HQ, UK; brambillar@cardiff.ac.uk; 3 School of Pharmacy and Pharmaceutical Sciences, Cardiff University, King Edward VII Avenue, Cardiff CF10 3NB, UK; brancalea@cardiff.ac.uk

**Keywords:** Ras, RasGRF1, hydrogen-bond surrogate, computational residue scanning, molecular dynamics, MM-GBSA, protein–protein interaction, ERK signalling, cocaine addiction, intellectual disability (ID), autism spectrum disorder (ASD)

## Abstract

In the last two decades, abnormal Ras (rat sarcoma protein)–ERK (extracellular signal-regulated kinase) signalling in the brain has been involved in a variety of neuropsychiatric disorders, including drug addiction, certain forms of intellectual disability, and autism spectrum disorder. Modulation of membrane-receptor-mediated Ras activation has been proposed as a potential target mechanism to attenuate ERK signalling in the brain. Previously, we showed that a cell penetrating peptide, RB3, was able to inhibit downstream signalling by preventing RasGRF1 (Ras guanine nucleotide-releasing factor 1), a neuronal specific GDP/GTP exchange factor, to bind Ras proteins, both in brain slices and in vivo, with an IC_50_ value in the micromolar range. The aim of this work was to mutate and improve this peptide through computer-aided techniques to increase its inhibitory activity against RasGRF1. The designed peptides were built based on the RB3 peptide structure corresponding to the α-helix of RasGRF1 responsible for Ras binding. For this purpose, the hydrogen-bond surrogate (HBS) approach was exploited to maintain the helical conformation of the designed peptides. Finally, residue scanning, MD simulations, and MM-GBSA calculations were used to identify 18 most promising α-helix-shaped peptides that will be assayed to check their potential activity against Ras-RasGRF1 and prevent downstream molecular events implicated in brain disorders.

## 1. Introduction

Maladaptive signalling mechanisms in the brain have been demonstrated in several psychiatric and neurological conditions. Amongst the most severe and poorly treated brain disorders, cocaine and psychostimulant addiction is a poorly managed chronic disease characterised by high relapse rates and compulsive drug use [1,2]. Most notably, addictive drugs exploit cellular mechanisms and signalling pathways involved in normal learning and memory processes [3,4,5,6]. Modulation of such learned associations between drug-paired cues and the rewarding effects of these drugs significantly contribute to persistently elicited drug-seeking behaviours and high rates of relapse [7,8,9,10,11,12,13,14,15,16,17,18,19,20,21,22]. The Ras (rat sarcoma protein)–ERK (extracellular signal-regulated kinase) pathway is crucially involved in both the acute and long-term effects of cocaine in experimental animal models, and previous work has shown that brain-penetrating Ras–ERK inhibitors may ameliorate the associated symptoms. Particularly relevant was the observation that Ras-RasGRF1 (Ras guanine nucleotide-releasing factor 1) interaction is responsible for the activation of the ERK cascade downstream of neurotransmitter receptor systems. Indeed, we previously showed that a cell-penetrating peptide, RB3, able to attenuate Ras-RasGRF1 binding could reduce downstream ERK signalling in response to cocaine in a mouse model. Similarly, RB3 was shown to ameliorate cellular effects and behavioural symptoms in two distinct mouse models of intellectual disability (ID) and autism spectrum disorder (ASD) [23,24].

**Figure 1:**
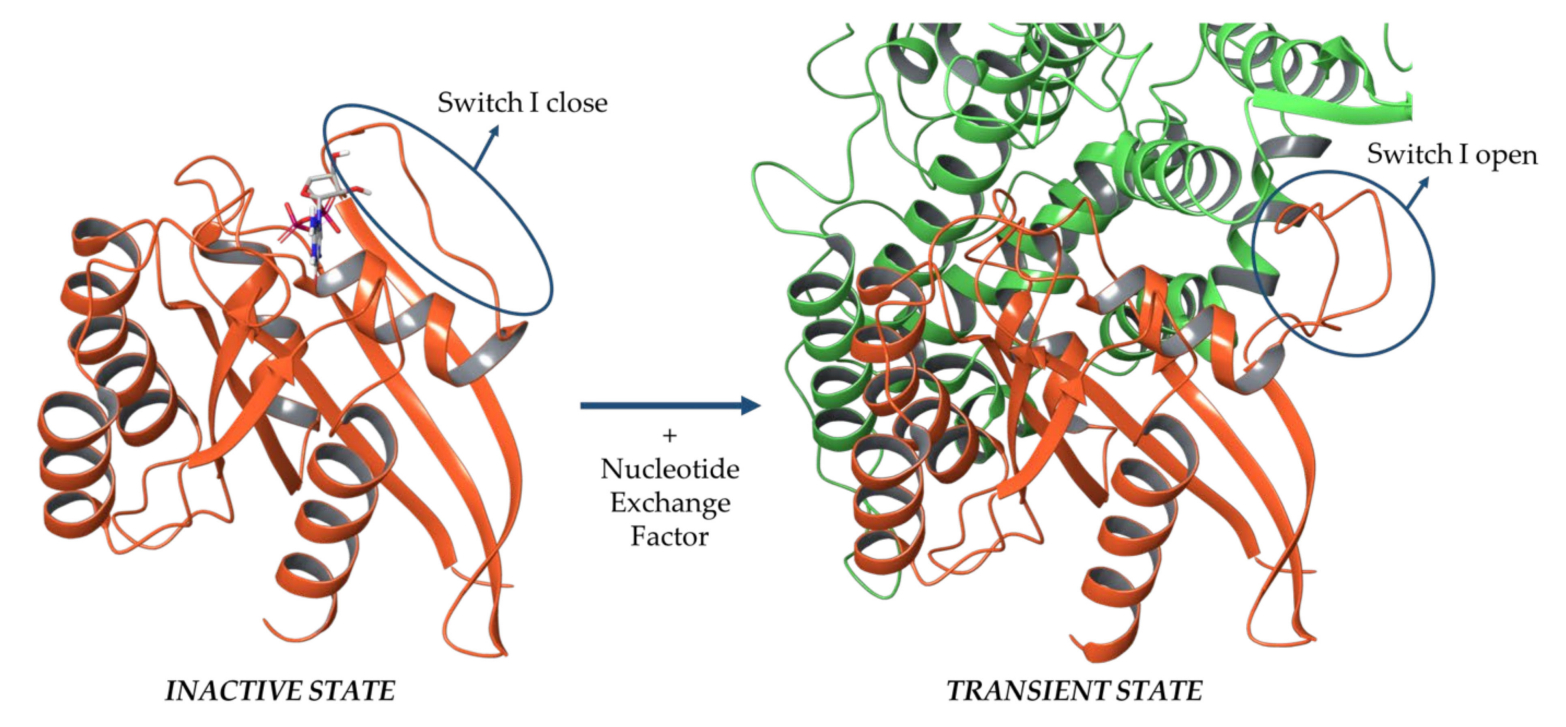
Inactive and transient states of the Ras protein. On the left, a Ras protein (orange chain; PDB ID: 1XD2 [48]) bound to GDP (grey ligand in stick format) exhibits its inactive state, where the Switch I region (highlighted by a blue circle) is closed; on the right, after binding to a guanine nucleotide-exchange factor (Sos protein, green chain), Ras (orange chain; PDB ID: 1XD2) exhibits a transient state, where the Switch I region is open to accommodate the GEF α-helix.

ERK signalling in most tissues responds to extracellular signals and regulates cell proliferation, differentiation, and survival [25,26,27]. In this context, Ras proteins act as binary switches in signalling pathways by cycling between inactive GDP- and active GTP-bound states [28]. Kinetic studies highlighted that the activation of Ras protein, proceeding from the conversion of Ras-GDP to Ras-GTP, initiates through the recruitment of the guanine nucleotide exchange factors (GEFs), such as RasGRF1 and Sos (Son of sevenless protein) [29,30,31,32,33,34], that catalyse GDP release and allow its replacement by GTP [35,36,37,38,39,40]. Then, the GTP molecule binds to this complex, promoting the release of the GEF protein [41]. The RasGRF1 and Sos region responsible for Ras-specific nucleotide exchange activity exhibits a Ras exchanger motif (Rem) domain of about 450 amino acids and a Cdc25 homology domain [42,43,44,45,46]. In addition, Sos requires allosteric activation through a second Ras-binding site that bridges the Rem and Cdc25 domains [47,48]. When Sos is activated, a helical hairpin belonging to the Cdc25 domain inserts between two flexible regions of Ras, switch I (amino acids 25–40) and switch II (amino acids 57–75) [49,50,51,52,53,54,55,56,57,58], causing Ras conversion to the transient state by opening the nucleotide-binding site of Ras for GDP release [43] (Figure 1). After this event, Ras can promptly accommodate and bind GTP into the nucleotide-binding site, thus exhibiting its active state. Therefore, a potential strategy to inhibit Ras-GEF interaction should target the open—or transient—state of Ras protein by designing modulators able to bind the nucleotide-exchange region.

In contrast to Sos, which requires Ras binding to the allosteric site for activity, the Cdc25 domain of RasGRF1 is active on its own [33,41,47,48]. The structure of the Cdc25 domain of RasGRF1 is very similar to that of Sos, registering 30% of sequence identity between the two Cdc25 domains. The orientation and conformation of the RasGRF1 helical hairpin resemble that of Sos in its active form, with an RMSD value of 2.3 Å for the helical hairpins after superposition on the Cdc25 domain core. Moreover, distance difference matrices demonstrated that the main differences between RasGRF1 and Sos in its inactive form have been identified in the helical hairpin, even in this case confirming that the RasGRF1 Cdc25 domain is more similar to active Sos [59]. Therefore, the analysis of the Ras-Sos complex might provide crucial insights, even for RasGRF1 interaction.

### 1.1. Mutational Studies on Sos and Design of a Peptide-Based Ras Inhibitor

To date, no complex structure of Ras-RasGRF1 is available in the Protein Data Bank [60]; thus, provided that Sos and RasGRF1 proteins share the Ras-specific nucleotide exchange domain [59], Ras-Sos X-ray crystallographic complexes were exploited for a comparative analysis.

Over the last decades, several mutational studies were conducted on Ras and Sos proteins to determine the key amino acids. In 1998, Boriack-Sjodin et al. [43] demonstrated that the contacts between Ras and Sos are mainly mediated by the Switch I and Switch II regions of Ras [43,49,50,51,52,53,54,55,56,57,58] and are essentially hydrophobic, polar, and charge–charge contacts. Hall et al. [61] performed site-directed mutagenesis to deeply investigate these contacts. The results shed light on the hydrophobic pocket of Sos protein, consisting of residues Ile825, Leu872, and Phe929, which embed the side chain of Tyr64 of Ras through hydrophobic contacts. In addition, the contribution of Tyr64 of Ras was explored by applying a mutation to alanine (Y64A). The result was a 50-fold reduction in the apparent binding affinities of Ras to Sos, but did not provide significant nucleotide dissociation. Then, the authors performed another binding assay by using wild-type (WT) Ras and mutated Phe929 of Sos to alanine (F929A). The Sos mutant reported a decrease of more than 50-fold in binding affinity for Ras. This data indicated that Tyr64 and Phe929 mediated crucial contacts for the formation of a stable Ras-Sos complex.

On the other hand, polar and charged interactions showed to be not essential for the binding affinity of Ras to Sos. Indeed, alanine mutations on Sos residues Arg826, Thr935, and Glu1002 weakly impacted on Ras binding and activation. In addition, the mutation of Ala59 of Ras to glycine (A59G) did not significantly affect the GDP-dissociation rate, displaying more than 50% of the inhibitory effect on Sos-catalysed GTP dissociation. Finally, the contribution of two Ras amino acids involved in the Switch I region were investigated: Tyr32 of Ras that established hydrophobic contacts with Lys939 of Sos, and Tyr40 of Ras that mediated stacking interaction with His911 of Sos. Tyr32 and Tyr40 of Ras were mutated to Ser (Y32S) and Ala (Y40A), respectively. Both mutations decreased the binding of Sos to Ras and accelerated the rate of intrinsic GDP/GTP exchange, suggesting that these residues are important for Ras-Sos recognition and the nucleotide stabilization. Consistent with these results, mutations of Sos Lys939 and His911 to alanine (K939A and H911A, respectively) also caused a reduction in Ras-Sos binding. Furthermore, the Y40A mutation had no significant effect on Sos-catalysed guanine nucleotide exchange, whereas the disruption of the contact between Tyr32 of Ras and Lys939 of Sos reduced the sensitivity of Ras to the exchange activity of Sos [43].

Several efforts have been reported in the literature to design and identify Ras inhibitors to block the nucleotide exchange. However, to date, the current scientific insights on ERK signalling in drug addiction has not been transferred into clinical treatments due to the lack of drugs with relatively low IC_50_ values, toxicity, and ability to efficiently cross the blood–brain barrier (BBB) [62].

In 2016, Papale and colleagues [63] designed and generated an active cell-penetrating peptide, named RB3 [64], based on the interaction between Ras and a GEF protein; i.e., RasGRF1, able to attenuate cocaine-mediated activation of the Ras–ERK signalling cascade in vivo. Subsequently, the same peptide was successfully used, in combination with the KIM sequence containing RB1 peptide, to treat two genetic animal models of intellectual disability and autism spectrum disorder, both characterised by an abnormally high ERK signalling activity [23,24].

The cell-penetrating peptides have been shown to be promising for the treatment of neuropsychiatric disorders, especially due to their low reported toxicity and tolerability [65,66]. Although their biological activity spans a micromolar range, they usually show a potential advantage, as they are able to partially disrupt protein–protein interactions without preventing the enzymatic activity. 

**Figure 2:**
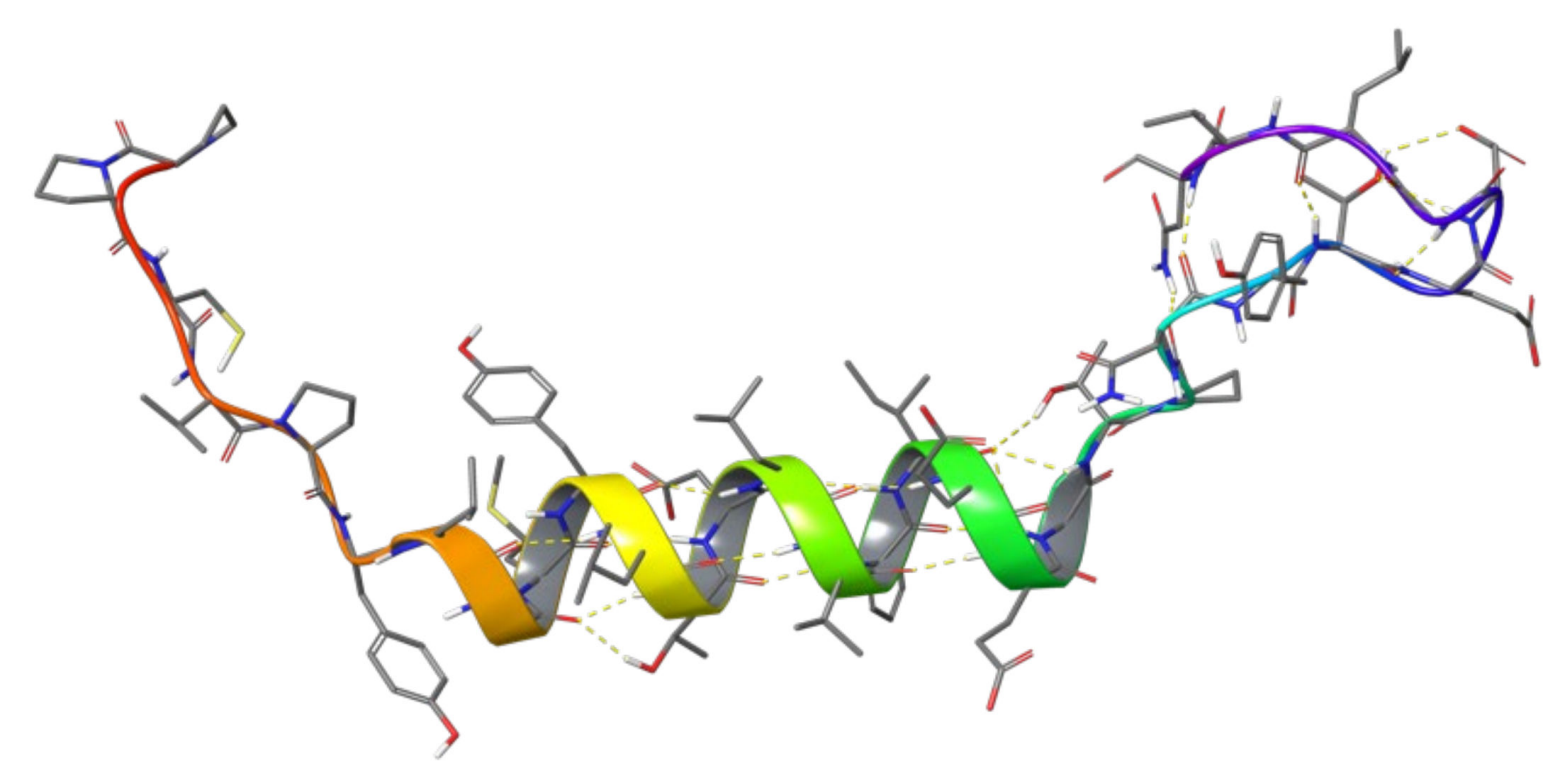
RB3 peptide structure including a loop from Pro1173 to Gly1180, an α-helix from Met1181 to Glu1191, and another loop from Gly1192 to Asn1203.

The RB3 peptide was designed by using molecular graphics tools, on the basis of the ternary complex consisting of Ras in its transient state bound to the Sos Cdc25 domain and Ras in its inactive state complexed with a GDP molecule (PDB ID: 1XD2) [48]. The Cdc25 domain of Sos involved in this ternary complex was compared to the crystal structure of the RasGRF1 Cdc25 domain (PDB ID: 2IJE [59]). The peptide sequence (amino acids 1173–1203 of the Cdc25 domain) includes an α-helix—from Met1181 to Glu1191—crucial for the GDP exchange activity on Ras proteins, linked to two loops—from Pro1173 to Gly1180 and from Gly1192 to Asn1203 (Figure 2).

Moreover, Papale and colleagues [67] added to the RB3 peptide sequence a portion retrieved from the HIV TAT protein known to exhibit a translocating behaviour [68]. In this way, the final structure of the cell-penetrating peptide was created able to cross the cell membranes and the BBB [67]. Thus, the peptide sequence is below reported:

GRKKRRQRRR—PPCVPYLGMYLTDLVFIEEGTPNYTEDGLVN
TAT sequence RasGRF1 interacting region

Then, the RB3 peptide was tested in an ex vivo model of acute striatal brain slices to investigate its inhibitory potential on ERK phosphorylation after stimulation with 100 µM of glutamate. The result was a significant reduction of ERK activity, with an IC_50_ of 6 µM. To deeply explore the effect of the RB3 peptide on Ras–ERK signalling pathway, Papale and colleagues investigated whether RB3 may also affect the phosphorylation of two well-characterised ERK substrates, (Ser10)-acetylated (Lys14) histone H3 (pAc-H3) and S6 ribosomal protein (pS6, Ser235/236 specific site) [69,70,71]. Even in this case, the peptide was effective in decreasing the phosphorylation of Ac-H3, with an IC_50_ of 5.2 µM; and pS6 levels, with an IC_50_ of 3.69 µM [63].

### 1.2. RB3 Peptide Modifications by Using Hydrogen-Bond Surrogates

**Scheme 1:**
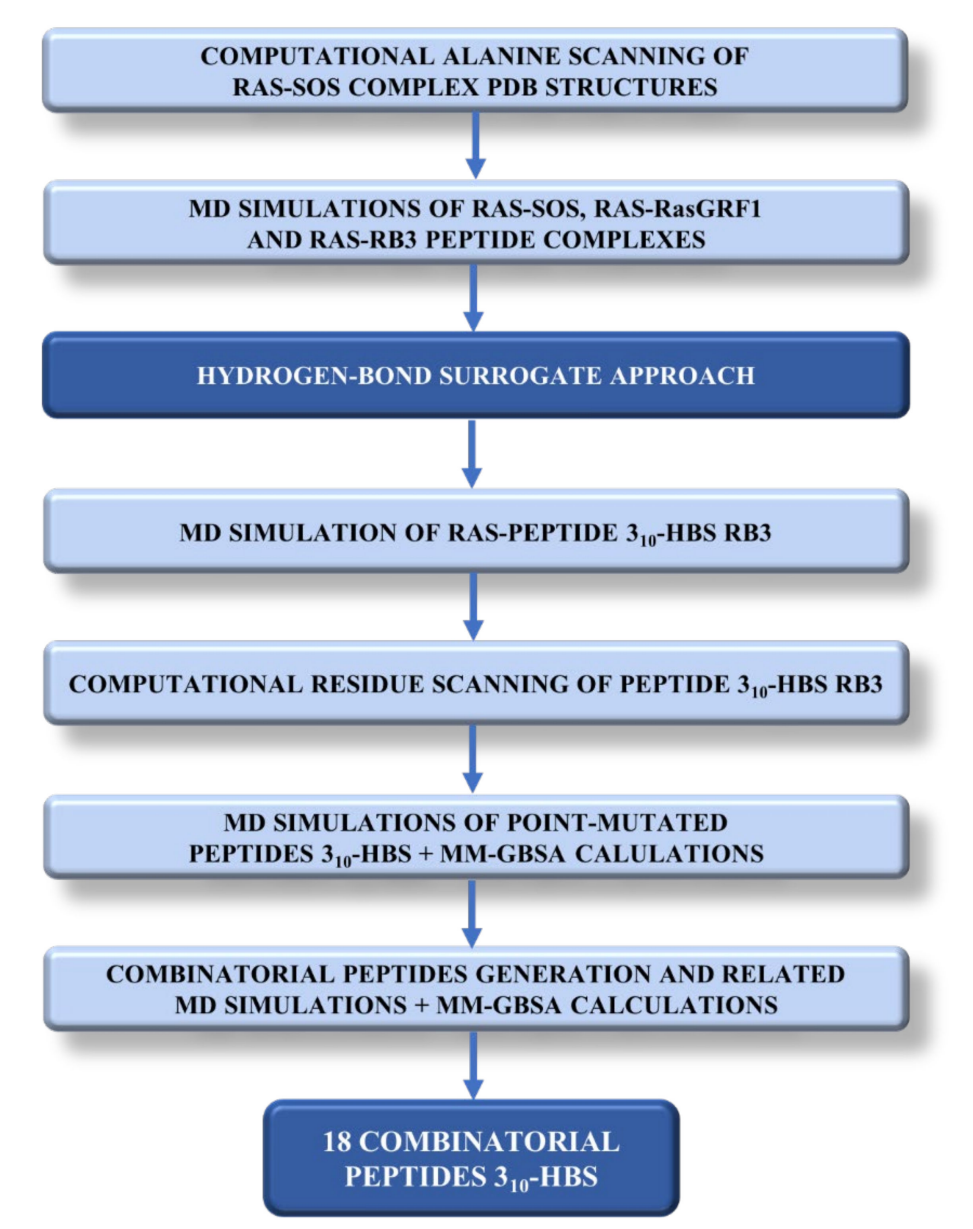
Overview of the computational workflow performed to identify potential peptide-based modulators to inhibit Ras-RasGRF1 interaction.

In light of the above, the RB3 peptide was selected to enter a compound optimisation process to increase the biological activity. This work aimed to provide insights on Ras-RasGRF1 interaction and suggest novel potential modulators of this interaction based on the RB3 peptide structure, which will be further investigated through biological assays. For this purpose, multiple computational techniques were exploited to investigate modifications of the RB3 peptide in order to potentially increase its inhibitory activity of Ras-RasGRF1 interaction. Scheme 1 lists the steps of the workflow that are described in detail in the following sections. 

First, a computational alanine scanning of the Ras-Sos complex available from the Protein Data Bank [60] was performed and, in parallel, molecular dynamics (MD) simulations on the three complexes (Ras-Sos, Ras-RasGRF1, and the Ras-RB3 peptide), were run to identify the most stable and frequent interactions between protein partners. Surprisingly, the RB3 peptide exhibited helicity loss, where the helical hairpin corresponding to RasGRF1 interacting region lost helicity propensity, generating instability within the complex. In order to optimise the structure of this peptide and increase the inhibitory capacity of the peptide, from the analysis of the literature, a potential strategy arose to solve this issue. Indeed, in the literature, similar cases of helicity loss have been reported and faced by exploiting the hydrogen-bond surrogate (HBS) approach [72]. This methodology was developed especially for modulating biomolecular interactions, such as protein–protein contacts, through small-molecular-weight protein secondary structure mimetics, when designing small molecules could be a very challenging strategy [73,74,75,76,77,78,79]. The HBS approach is based on the helix-coil transition theory for peptides, whereas α-helices composed of a few amino acids are expected to be essentially unstable due to a low nucleation probability [80,81]. This approach is expected to overwhelm the intrinsic nucleation propensities of the amino acids by providing a preorganization of the residues upstream, which triggers the helix formation initialization [82,83]. An example of a successful case of HBS use was the HBS3 peptide [62,84], a synthetic α-helix that reduced the Ras nucleotide exchange in vitro and modestly activated ERK in cells [62,84]. This peptide was basically built on the Sos sequence able to bind the Ras protein, and incorporates the HBS strategy reported in the literature [62,72,84]. On the other hand, in the literature, there is no evidence that the HBS methodology has been applied to the RB3 peptide. Thus, it appeared interesting to investigate the employment of this strategy aiming at avoiding the helicity loss of RB3 peptide.

In general, in an α-helix, the carbonyl group of the *i*th amino acid residue mediates a hydrogen bond with the amine group of the (*i* + 4)th amino acid residue by generating nucleation and stabilisation of the helical structure. Based on this evidence, the HBS strategy for generation of artificial α-helices involves the replacement of one of the main chain hydrogen bonds with a covalent linkage [73,85]. Indeed, to mimic the C=O · · · H-N hydrogen bond as closely as possible, a covalent bond of the type C=X–Y–N is included, where X and Y are usually carbon atoms that would be part of the *i*th and the (*i* + 4)th residues. However, the analysis of the RB3 α-helix highlighted that the first amino acid implicated in the helix H-bond ensemble does not establish a traditional hydrogen bond with the (*i* + 4)th amino acid, while it forms a contact with the (*i* + 3)th amino acid by creating the so-called 3_10_-helix [86]. Therefore, in this work, the RB3 peptide was modified by creating a C-C bond between the first (Tyr1178) and the fourth amino acid (Met1181), hereafter called the 3_10_-HBS RB3 peptide. An MD simulation of the complex Ras-3_10_-HBS RB3 peptide was run, highlighting a stable peptide helical conformation during the entire trajectory.

Then, a computational residue scanning was run on the peptide to analyse and identify the most promising mutations to be considered in terms of ΔΔG_affinity_ and ΔΔG_stability_. The point-mutated peptides, in complex with the Ras protein, were exploited to run MD simulations and MM-GBSA calculations to guide the selection of the most interesting mutations. The resulting ones were further combined with each other in the 3_10_-HBS RB3 peptide structure to obtain 48 combinatorial peptides. Thus, these latter were investigated through MD and MM-GBSA to retrieve the calculated ΔG_binding_ average values for each couple Ras-combinatorial peptide. Finally, 18 combinatorial peptides were selected, and they will enter an experimental follow-up to further explore their potential activity against the Ras-RasGRF1 interaction. In the next sections of this manuscript, efforts to modify the peptide structure to increase the biological activity will be described.

## 2. Results and Discussion

### 2.1. Sos and RasGRF1 Binding Interfaces Analysis

To date, no PDB structures of the Ras-RasGRF1 complex are available in the literature, thus the Ras-Sos complex structure was exploited to collect key information useful to guide and address the computational studies described herein. In order to deeply explore and predict the hot-spot residues of the Sos Cdc25 domain (i.e., amino acids 924 to 957), all of the six available PDB structures of the Ras-Sos complex (PDB IDs: 1XD2 [48], 1BKD [43], 1NVW, 1NVV, 1NVU, and 1NVX [47]) were examined by using the Robetta Computational Interface Alanine Scanning Server [87,88]. In Table 1, the highest ΔΔG values from the alanine mutations on the Sos binding interface are reported.

These predicted data were in accordance with mutational studies performed by Hall et al. [61], whereas Phe929, Thr935, and Lys939 were highlighted as Sos interacting hot spots. In detail, from the computational alanine scanning, Phe929 and Thr935 were shared by most of the six PDB complexes as hot spots, while Lys939 resulted from the PDB 1NVV analysis. As can be seen, this computational tool pointed out another Sos hot spot not previously identified by Hall et al., Asn944, which was shared from all the six PDB complexes. Another hot spot on Glu942 was retrieved from PDB 1NVW. 

These identified hot spots, both from biological assays [61] and computational alanine scanning, were considered equally important for the next steps, and were used for comparison to RasGRF1 amino acids in order to investigate similarities between the two GEF sequences (RasGRF1 and Sos). For this purpose, PDB 1XD2 [48], including Sos in complex with the Ras protein, was chosen for the low resolution, while the only available PDB structure of the RasGRF1 Cdc25 domain (PDB ID: 2IJE [59]) was used. However, the latter PDB was from *Mus musculus* as organism. Hence, before proceeding with the protein structure alignment between the Sos and RasGRF1 Cdc25 domains, a FASTA alignment was performed while considering human and murine RasGRF1 sequences through the Protein BLAST sequence alignment tool [89,90]. The resulted overall identity was 83.22%, whereas within the RasGRF1 region involved in Ras binding (i.e., from residue 1173 to 1203 of mouse sequence), the only detected difference was between Ala1198 for human and Val1187 for mouse, as illustrated in Figure S1 in the Supplementary Materials. These two amino acids exhibited side chains with very similar chemical properties, thus the PDB 2IJE was considered suitable for proceeding with this study.

**Figure 3:**
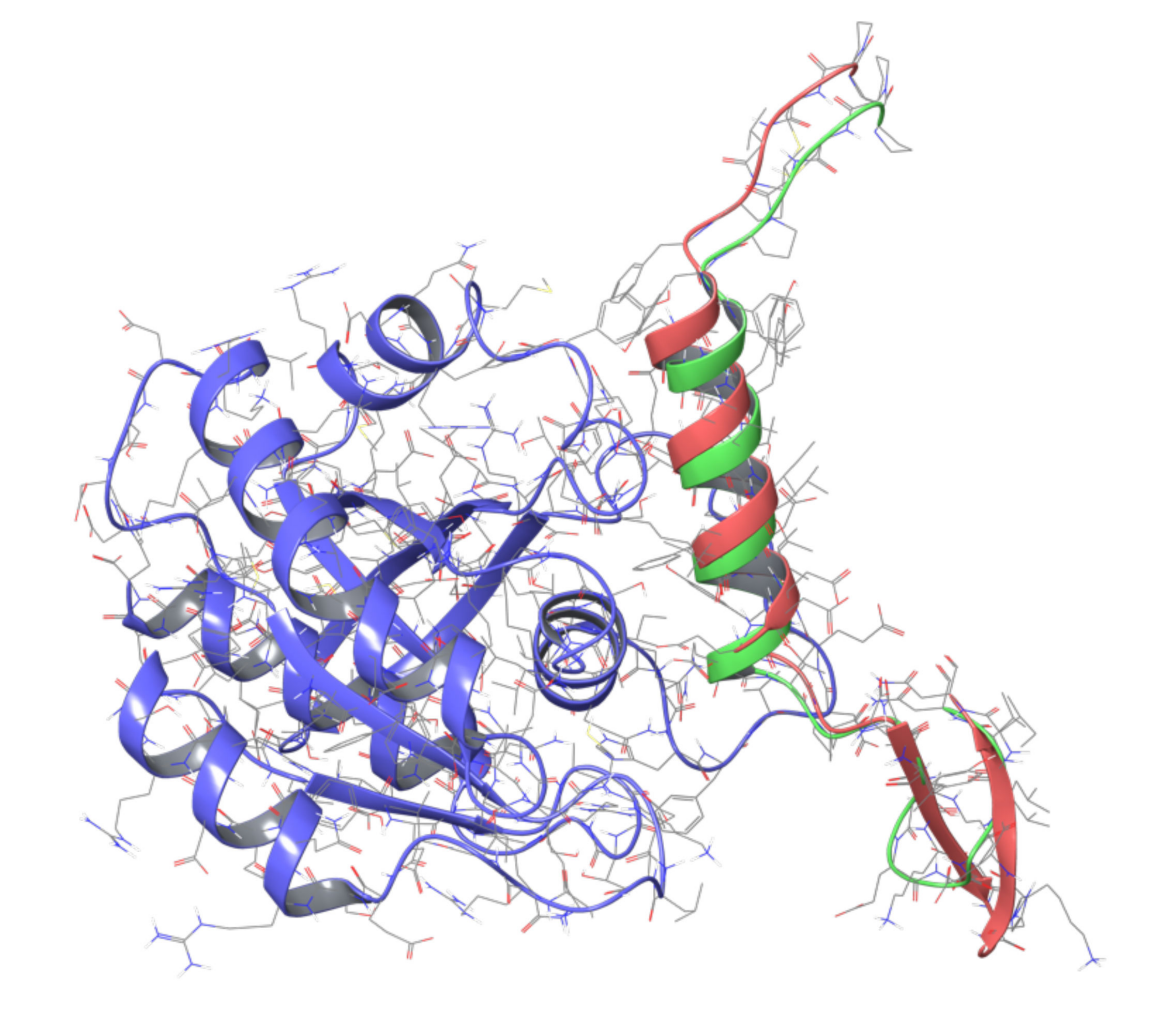
Superposition of the RasGRF1 binding region (green chain, retrieved from PDB 2IJE [59]), and the Sos binding region (pink chain, retrieved from PDB 1XD2 [48]) in complex with the Ras protein (blue chain from PDB 1XD2).

Therefore, both PDB protein structures (2IJE [59] and 1XD2 [48]) were aligned through the “Protein Structure Alignment” tool of the Schrödinger suite, and the result is depicted in Figure 3. As can be seen, the two α-helices of Sos and RasGRF1 are perfectly aligned.

**Figure 4:**
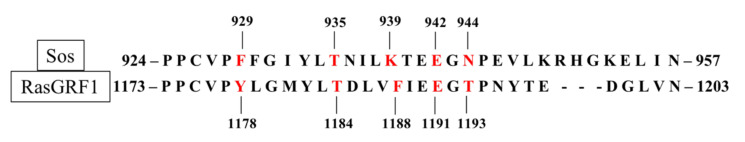
FASTA sequence alignment between the Sos and RasGRF1 regions able to bind the Ras protein.

Furthermore, Sos and RasGRF1 binding regions share several amino acids, as shown in the sequence alignment of the Sos and RasGRF1 Cdc25 domains illustrated in Figure 4.

RasGRF1 amino acids corresponding to Sos hot spots are reported in Table 2. As can be seen, the pairs Thr935-Thr1184 and Glu942-Glu1191 shared the same amino acid, while Phe929 (Sos) and Tyr1178 (RasGRF1) both presented hydrophobic side chains, and Asn944 (Sos) and Thr1193 (RasGRF1) shared polar uncharged side chains. Only Lys939 and Phe1188 were very different amino acids, whereas lysine showed an electrically charged side chain, while phenylalanine exhibited a hydrophobic side chain. The amino acids of RasGRF1 highlighted in the above-described comparison were considered for the next steps of the analysis.

### 2.2. MD Simulations of Ras in Complex with Sos and RasGRF1 Binding Fragments

In order to investigate the importance of the computationally predicted hot spots not reported in the literature; i.e., Glu942 and Asn944, and to explore the interactions between the Ras-Sos complex, a MD simulation was run. For this purpose, PDB 1XD2 [48] was chosen, and the simulation was run for a short time of 50 ns to observe and identify the most stable and frequent contacts. The stability of the system was monitored during the entire trajectory, thus registering the RMSD plot depicted in Figure S2 in the Supplementary Materials. Then, the MD frames were clustered using the “Desmond trajectory clustering” tool of the Schrödinger suite (Schrödinger Inc., New York, NY, USA, software release v2018-4) by setting five clusters to be generated, and the frame centroids representative for the clusters were analysed; i.e., frame 120, frame 540, frame 660, frame 780, and frame 820. These five frames were investigated to identify the interactions between the Ras and Sos proteins, where among the above-mentioned five Sos hot spots (please refer to Table 2), four residues established stable interactions with Ras during the trajectory; i.e., Phe929, Thr935, Glu942, and Asn944. The related interactions are listed in Table 3, and the H-bonds are plotted against the simulation time in Table S1 in the Supplementary Materials.

Based on the previously described comparison between the Sos interacting region and the RasGRF1 Cdc25 domain, an MD simulation of the Ras-RasGRF1 complex was performed through the Schrödinger suite [91] to investigate whether the five putative RasGRF1 key residues (Tyr1178, Thr1184, Phe1188, Glu1191, and Thr1193) were responsible for contacting the Ras protein and stabilising the complex.

Therefore, the protein–protein complex was generated by using the previous aligned structures involving PDBs 1XD2 [48] and 2IJE [59], where the RasGRF1 interacting region (residues 1173 to 1203) was located within the binding pocket of the Ras protein through performing a superimposition on the Sos chain, which was subsequently deleted. The complex was minimised, and the MD was run by setting 50 ns as the simulation time. The output was analysed, and the stability of the system was checked through the RMSD plot (Figure S3 in the Supplementary Materials). The trajectory was clustered by setting five clusters to be generated. Then, the MD frame centroids were analysed; that is, frame 40, frame 65, frame 110, frame 280, and frame 362. The most stable interactions between Ras and RasGRF1 were observed, and they are reported in Table 3. In Table S2 in the Supplementary Materials, the H-bonds are plotted against the simulation time. As can be observed, most of these interactions were similarly established between Ras and the corresponding Sos amino acids (please refer to Table 2) during the MD simulation. This provided interesting information to take forward in this work.

### 2.3. MD Simulations of the Ras-RB3 Peptide Complex

After collecting information about interactions between Ras and its GEFs (Sos and RasGRF1), other MD simulations were performed to explore the binding mode and the established contacts between Ras and the parental (WT) RB3 peptide. 

The core sequence of this peptide (without TAT portion) corresponds to RasGRF1 sequence 1173-PPCVPYLGMYLTDLVFIEEGTPNYTEDGLVN-1203. Therefore, the complex Ras-RasGRF1 was used, and all those residues not included in the RB3 peptide sequence were deleted. Thus, this new complex, the Ras-RB3 peptide, was processed by running MD simulations of 500 ns each. The RMSD plot was generated, and it is depicted in Figure S4a in the Supplementary Materials. This plot revealed a certain instability of the systems, ranging from about 3.5 to 6.4 Å. Therefore, a second MD simulation was computed, and even in this case the RMSD plot (Figure S4b in the Supplementary Materials) showed the same trend. On the other hand, the interactions between Ras and the peptide were investigated for both trajectories. The MD frames of both simulations were grouped into five clusters each, and the frame centroids were analysed to retrieve the most stable and frequent interactions: (a) first MD → centroid frame 390, centroid frame 2340, centroid frame 1400, centroid frame 3590, and centroid frame 4270; (b) second MD → centroid frame 3610, centroid frame 4800, centroid frame 2660, centroid frame 620, and centroid frame 1280. The observed contacts between the Ras protein and the RB3 peptide from the above-listed frames are reported in Table 4.

The two MD simulations shared only two interactions; i.e., one hydrogen bond between Tyr40 of Ras and Asp1185 of the RB3 peptide, and a pi–pi stacking between the aromatic ring of the Tyr40 side chain of Ras and the other aromatic ring of the Phe1188 side chain of the RB3 peptide. Furthermore, all the other interactions retrieved from the MD simulations registered low stability during the entire trajectories.

**Figure 5:**
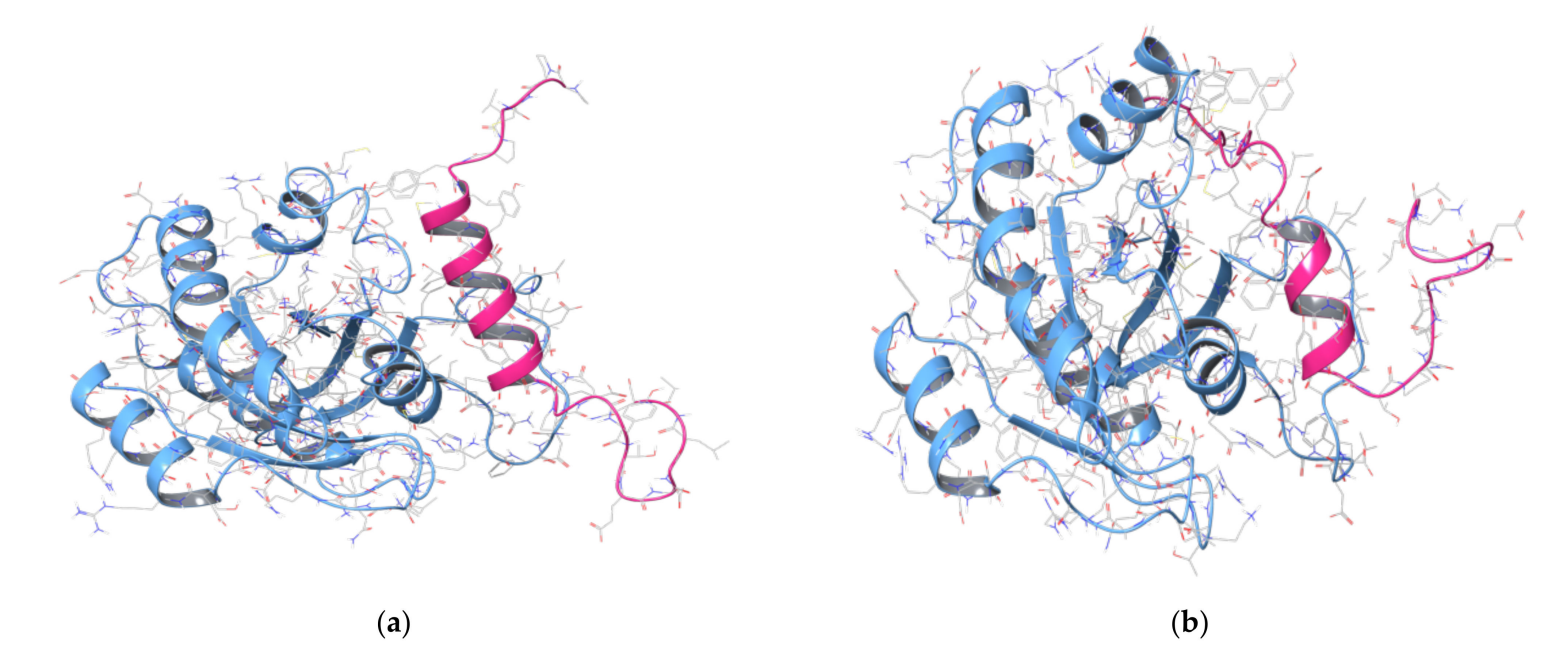
(**a**) Frame 0 of the first MD simulation on Ras (blue chain) in complex with a RasGRF1 fragment (pink chain, aa 1173 to 1203); (**b**) frame from the first MD simulation on Ras in complex with a RasGRF1 fragment after about 50 ns, depicting the RB3 peptide losing helicity propensity in the portion from Met1181 to Thr1184.

Thus, a visual check of both simulations shed light on an important behaviour of the RB3 peptide α-helix; i.e., a portion of the α-helix (from Met1181 to Thr1184) began to lose helicity propensity after about 50/60 ns of simulation time, resulting in a misfolding behaviour. Indeed, not surprisingly, the two above-mentioned interactions shared by both simulations from the MD analyses were involved into the folded region of the peptide over the entire trajectories; i.e., the residues from Asp1185 to Glu1191 not exhibiting the misfolding. Figure 5 illustrates the misfolding of the RB3 α-helix after 50 ns of the first MD simulation.

Therefore, a strategy to overcome this issue was implemented by applying the hydrogen-bond surrogate approach, which had already provided successful experimental evidence [62,73,74,75,76,77,78,79,84]. Thus, the RB3 peptide was processed by modifying the structure. First, two portions of the peptide were deleted; i.e., the residues belonging to the two loops of the peptide (residues 1171 to 1177 and 1194 to 1203), because they showed a lack of crucial interactions according to mutational studies reported in the literature [43,61] and the MD simulations of Ras-RasGRF1 (please refer to Table 3). Thus, residues 1171 to 1177 and 1194 to 1203 were considered not important for the purpose of this work, and were deleted from the structure. Then, the analysis of the α-helix highlighted that Tyr1178, the first amino acid implicated in the helix H-bonds ensemble, did not establish a traditional hydrogen bond with the (*i* + 4)th amino acid, while it formed a contact with the (*i* + 3)th amino acid, by creating the so-called 3_10_-helix [86].

**Figure 6:**
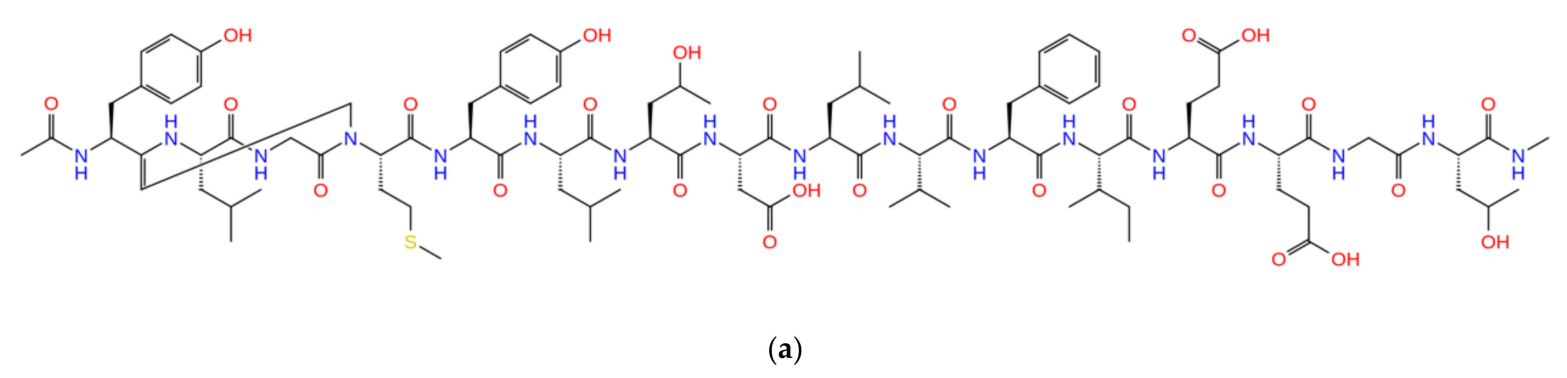

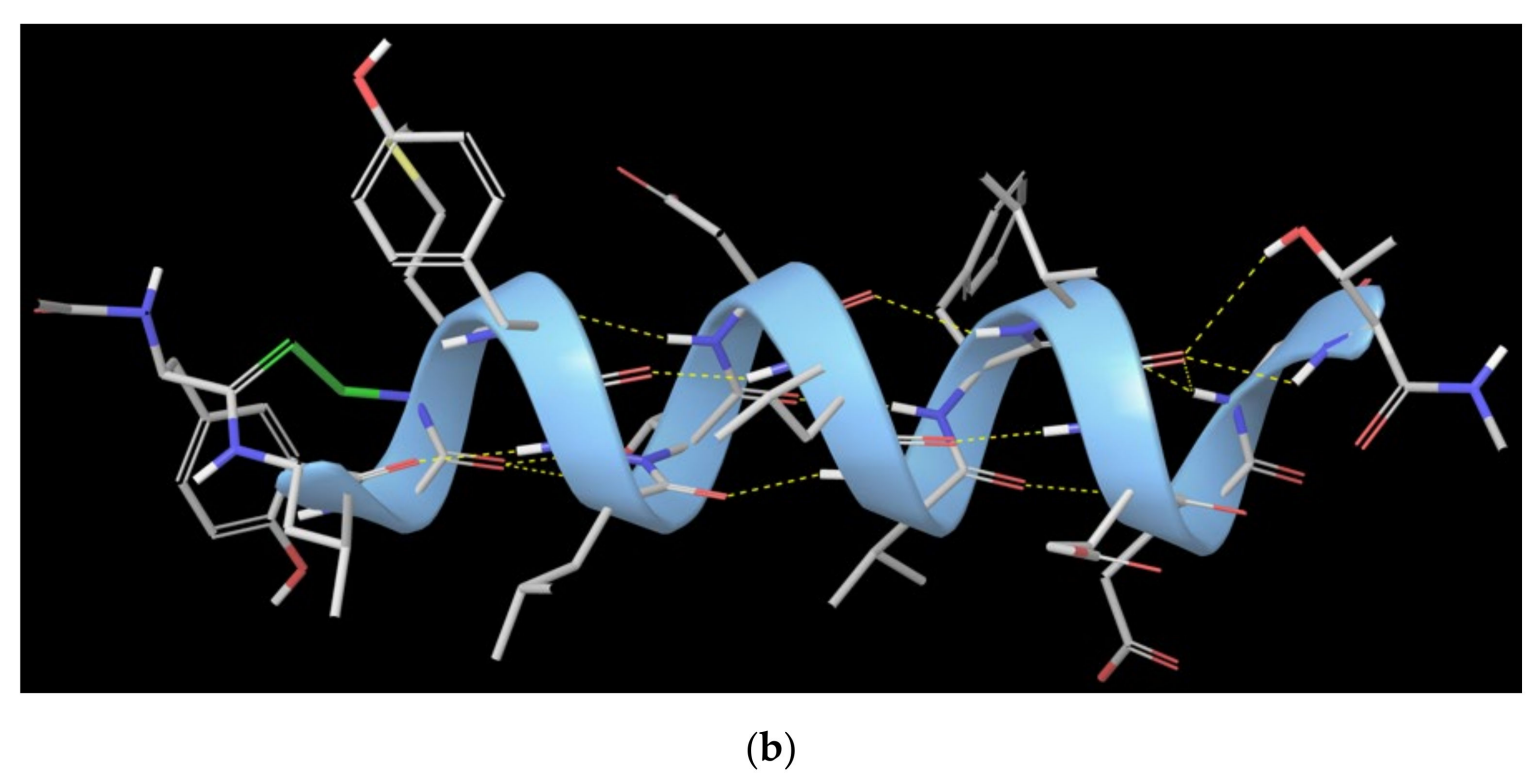
(**a**) Structure of the 3_10_-HBS RB3 peptide; (**b**) the 3_10_-HBS RB3 peptide including a covalent C-C bond (green bond) between the carbonyl oxygen of Tyr1178 backbone and the amine hydrogen of Met1181 backbone.

Hence, a MD simulation of 500 ns was performed on the Ras protein in complex with the RB3 peptide modified by deleting the two loops at the N- and C-termini and creating a covalent C-C bond between the carbonyl oxygen of the Tyr1178 backbone and the amine hydrogen of the Met1181 backbone. The resulting peptide was termed 3_10_-HBS RB3 (Figure 6).

**Figure 7:**
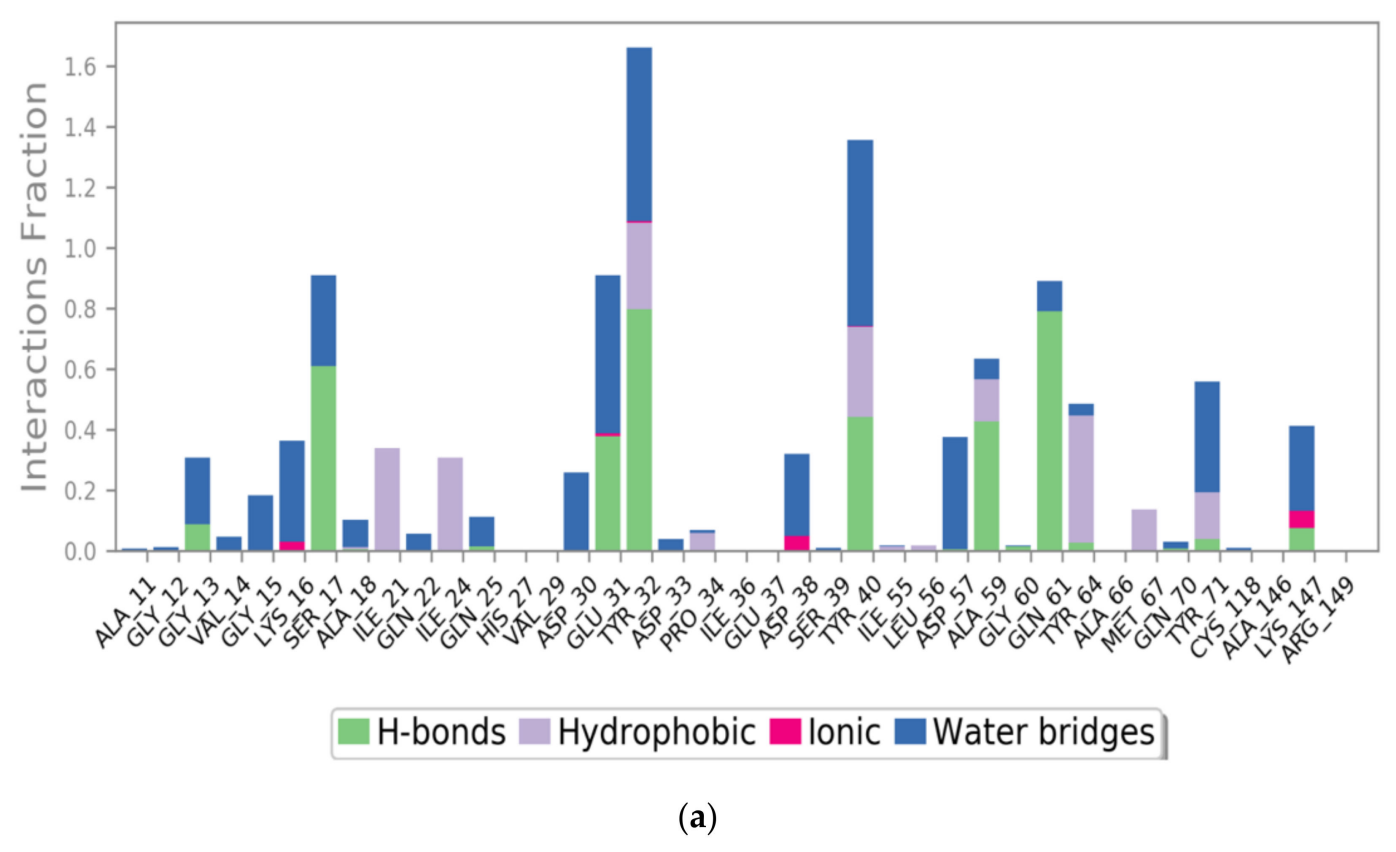

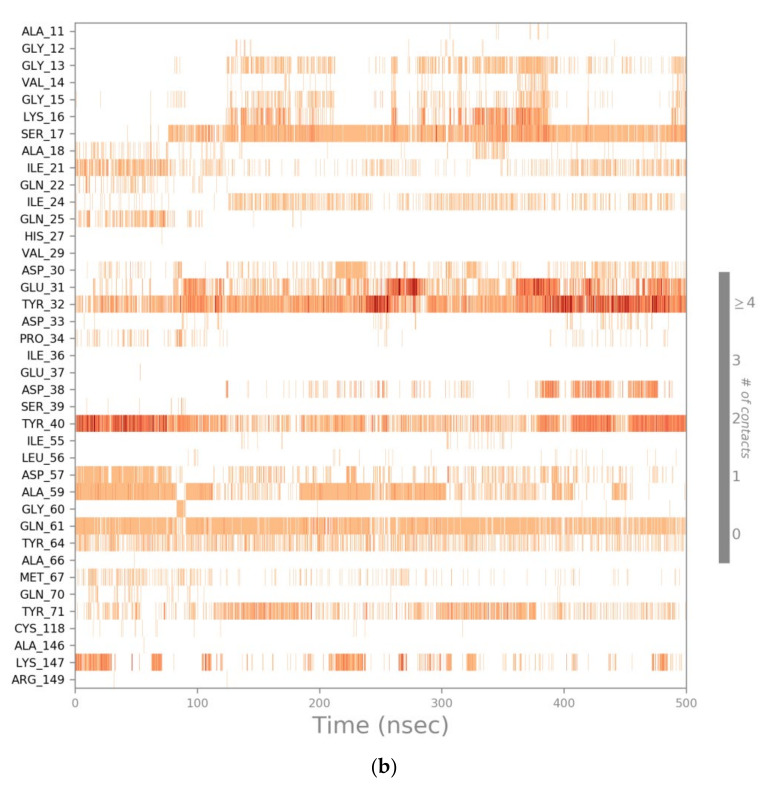
(**a**) Histogram of the interactions established between the Ras protein binding region and the 3_10_-HBS RB3 peptide; (**b**) Plot illustrating the frequency of interaction occurrences between the Ras protein binding region and the 3_10_-HBS RB3 peptide.

The analysis of the output revealed a stable trend for the α-helicity of the peptide, which held its folded conformation. Even the RMSD plot (Figure S5 in the Supplementary Materials) showed a certain stability of the system, thus the frames were analysed to retrieve information about the most stable interactions, and the results were plotted as depicted in Figure 7.

This newly designed 3_10_-HBS RB3 peptide showed that it could establish some of the key interactions identified in the previous MD simulation between the Ras and RasGRF1 proteins (please refer to Table 3) and other contacts with Ras amino acids (Tyr32 and Tyr40) highlighted as key residues from mutational studies [61]. Finally, an MM-GBSA calculation of the MD frame was computed to obtain the ΔG_binding_ of the complex Ras-3_10_-HBS RB3 peptide, which was −79.70 kcal/mol. This value was exploited as a reference for the peptide optimisation process described in the following sections.

### 2.4. Computational Residue Scanning of the 3_10_-HBS RB3 Peptide and MD Simulations of Point-Mutated Peptides

In order to optimise the structure of the 3_10_-HBS RB3 peptide, a computational residue scanning was performed on the amino acids of the peptide by using the “Residue scanning” tool of Bioluminate (Schrödinger Inc., software release v2018-4) [92]. The peptide was point-mutated where each residue was substituted with all the standard amino acids, and ΔΔG_affinity_ and ΔΔG_stability_ values of the new complexes were computed. The aim was to identify the most promising mutations in terms of ΔΔG_affinity_ and ΔΔG_stability_. For this purpose, only mutations reporting both ΔΔG_affinity_ and ΔΔG_stability_ values below −3 kcal/mol were considered, according to the work of Beard et al. [92], which reported a correlation between experimental results and the computationally predicted ones through the Schrödinger suite. Indeed, the authors demonstrated that a difference of 3 kcal/mol between the mutated and WT forms of a complex might be considered reliable in hot-spot prediction. Finally, 16 mutations reported ΔΔG_affinity_ and ΔΔG_stability_ values lower than −3 kcal/mol, thus they were considered for the next steps of this work (Table 5).

These 16 mutations were used to create as many complexes involving the Ras protein and the 3_10_-HBS RB3 point-mutated peptides that underwent MD simulations. The trajectory time was set at 100 ns for each system, since this timeframe was considered suitable to detect potential misfolding of the peptides. Indeed, the previously described MD simulations on the WT RB3 peptide exhibited misfolded conformation by losing α-helicity after about 50/60 ns of simulation. From the analysis of the MD trajectories, all the point-mutated peptides were able to keep the helical conformation, thus MM-GBSA calculations were computed, and the related ΔG_binding_ values are reported below in Table 6. Table S3 in the Supplementary Materials lists the ΔG_binding_ average values of the interaction energies and the generalized Born solvation energy for the MD trajectories of the complexes’ Ras-point-mutated 3_10_-HBS RB3 peptides. The stability of the systems was investigated by analysing the RMSD plots per each complex, resulting in suitable stationary shape for each system (Table S4 in the Supplementary Materials).

As previously mentioned, the ΔG_binding_ of the complex between Ras and the WT 3_10_-HBS RB3 peptide (−79.70 kcal/mol) was used as a reference to select the most promising mutations associated with ΔG_binding_ values lower than the reference one. In light of the above, from the MM-GBSA results, only three mutated peptides showed higher ΔG_binding_ values. Hence, the related mutations, F1188H, E1190H, and E1191I, were neglected. On the contrary, the other 13 mutations were considered for creating combinatorial peptides, as described in the next section.

### 2.5. Combinatorial Peptides Using 3_10_-HBS RB3: Creation and MD Simulations

The most promising mutations on the 3_10_-HBS RB3 peptide were combined with each other to obtain 48 mutated peptides overall, as listed below in Table 7.

The 48 combinatorial peptides in complex with the Ras protein were processed by running MD simulations of 100 ns each to investigate helix conformational stability and the contacts formed with the Ras protein. All the trajectories were observed by generating RMSD plots to ensure the reliability of the outputs, and the interaction frequency and stability were analysed. Finally, MM-GBSA calculations of the MD simulations were computed. Thus, all those combinatorial peptides not responding to the following criteria were neglected:

1. ΔG_binding_ value higher than the reference one (−79.70 kcal/mol);
2. Loss of helical conformation.

Finally, 18 combinatorial peptides overall fulfilled the above criteria by resulting in promising ΔG_binding_ values and exhibiting a helical trend during the whole MD trajectory. Thus, Table 8 reports the MM-GBSA results of these 18 most promising combinatorial peptides, which will be considered for the follow-up of this study by carrying out biological assays. Table S5 in the Supplementary Materials lists the ΔG_binding_ average values of the interaction energies and the generalized Born solvation energy for the MD trajectories of the complexes’ Ras-combinatorial peptides. The related RMSD plots and interaction diagrams of these 18 selected peptides are reported in Tables S6 and S7, respectively, in the Supplementary Materials. Based on the above-mentioned plots, these selected combinatorial peptides were able to mainly reproduce the key interactions of the 3_10_-HBS RB3 peptide reported above in Figure 7 by establishing contacts with Ras key residues, especially Tyr32, Tyr40, and Tyr64, which were highlighted as crucial by previous experimental assays [43,61]. 

**Figure 8:**
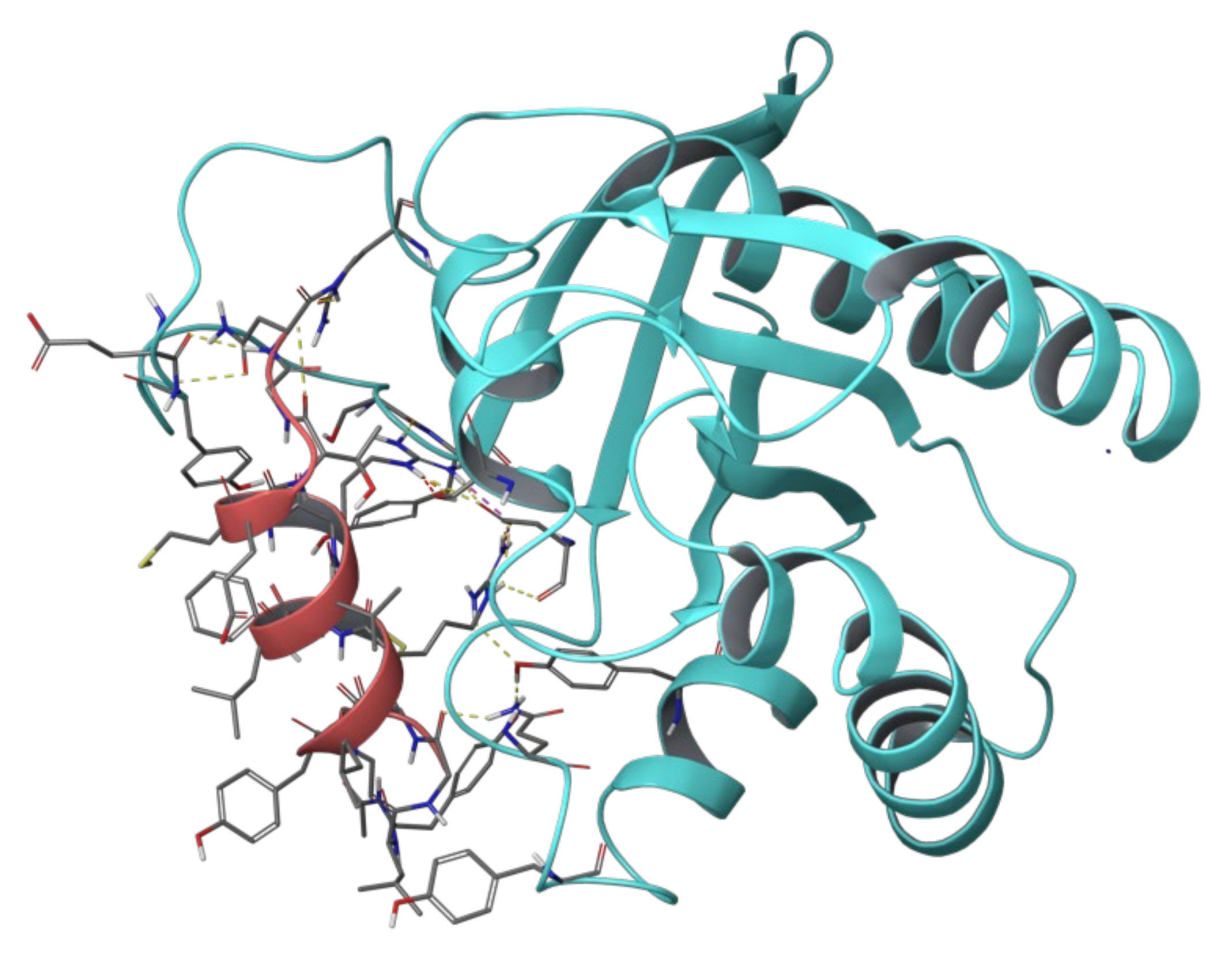
Frame of the MD simulation performed on the combinatorial peptide forty-three in complex with the Ras protein, depicting the binding mode of the peptide.

Furthermore, other contacts appeared, especially with Asp57, Gly60, and Gln61 of Ras. Indeed, these amino acids were involved in interactions with Sos and RasGRF1 according to previous MD simulations, thus confirming that these designed peptides might bind and inhibit the interaction between the Ras protein and the guanine nucleotide exchange factors, Sos and RasGRF1. Figure 8 depicts the binding mode of the 3_10_-HBS combinatorial peptide forty-three, which reported the lowest ΔG_binding_ average.

## 3. Methods

### 3.1. Protein Preparation

The 3D structures of the Ras-Sos complex (PDB IDs: 1XD2 [48], 1BKD [43], 1NVW, 1NVV, 1NVU, and 1NVX [47]) and RasGRF1 protein (PDB ID: 2IJE [59]) were retrieved from the Protein Data Bank [60] and optimised using the “Protein preparation” tool of the Schrödinger suite (Schrödinger Inc., New York, NY, USA) software release v2018-4) [93]. The bond orders for untemplated residues were assigned by using known HET groups based on their SMILES strings in the Chemical Component Dictionary. Hydrogens were added to the structure, zero-order bonds between metals and nearby atoms were added, and formal charges to metals and neighbouring atoms were corrected. Disulfide bonds were created according to possible geometries, and water molecules beyond 5.0 Å from any of the HET groups, including ions, were deleted. Then, protonation and metal charge states for the ligands, cofactors, and metals were generated [94,95]. Finally, PROPKA [95] was run under pH 7.0 to optimise hydroxyl groups and Asn, Gln, and His states.

### 3.2. MD Simulations of Ras Protein in Complex with Sos, RasGRF1, RB3 Peptide, and the Designed 3_10_-HBS Peptides 

In this work, 69 MD simulations were performed using Desmond [91,96,97,98,99], as follows: 1 MD simulation of 50 ns for the Ras-Sos complex, 1 MD simulation of 50 ns for the Ras-RasGRF1 complex, 2 MD simulations of 500 ns for Ras in complex with the WT RB3 peptide, 1 MD simulation of 500 ns for Ras in complex with the 3_10_-HBS RB3 peptide, 16 MD simulations of 100 ns for Ras complexed with the point-mutated 3_10_-HBS peptides, and 48 MD simulations of 100 ns for Ras in complex with the combinatorial 3_10_-HBS peptides. All the trajectories were computed by applying the same MD settings below described. The systems were created using TIP3P [100] as a solvent model, and the orthorhombic shape box was chosen. The box side distances were set at 10 Å. The force field OPLS3e [101] was applied, and the systems were neutralized by adding Na^+^ ions. The outputs were further processed by performing MD simulations with the above-reported simulation times.

The ensemble class NPT was chosen to maintain the number of atoms, the pressure, and the temperature constant for the entire trajectories. The thermostat method employed was the Nosé–Hoover chain with a relaxation time of 1.0 ps and a temperature of 300 K. The barostat method applied was Martyna–Tobias–Klein, with a relaxation time of 2.0 ps and an isotropic coupling style. The timestep for numerical integration was 2.0 fs for bonded interactions, 2.0 fs for nonbonded-near (van der Waals and short-range electrostatic interactions), and 6.0 fs for nonbonded-far (long-range electrostatic interactions). For Coulombic interactions, a cut-off radius of 9.0 Å was tuned as a short-range method. Pressure and temperature were set at 1.01325 bar and 300 K, respectively. Finally, the systems were relaxed before beginning the simulations according to the following steps:

1. Minimization with the solute restrained;
2. Minimization without restraints;
3. 12 ps in the NVT ensemble with a Berendsen thermostat, temperature of 10 K, a fast temperature relaxation constant, velocity resampling every 1 ps, and nonhydrogen solute atoms restrained;
4. 12 ps in the NPT ensemble in a Berendsen thermostat and barostat, temperature equal to 10 K and a pressure of 1 atm, a fast temperature relaxation constant, a slow pressure relaxation constant, velocity resampling every 1ps, and nonhydrogen solute atoms restrained;
5. 24 ps in the NPT ensemble with a Berendsen thermostat and barostat, temperature of 300 K and a pressure of 1 atm, a fast temperature relaxation constant, a slow pressure relaxation constant, velocity resampling every 1 ps, and nonhydrogen solute atoms restrained;
6. Final step of 24 ps of relaxation in NPT ensemble using a Berendsen thermostat and barostat, a temperature of 300 K and a pressure of 1 atm, a fast temperature relaxation constant, and a normal pressure relaxation constant.

### 3.3. MD Frame Clustering

In order to retrieve the key contacts between the protein partners during the entire simulations, for the MD simulations performed for Ras-Sos, Ras-RasGRF1, and Ras-RB3 peptide complexes, the frames were clustered to identify the most representative centroids to be analysed. The RMSD matrix calculation was set using the protein backbone as reference, the frequency of frames analysis was set 10, and the hierarchical cluster linkage method as average. Finally, for each MD trajectory, five clusters were generated; the analysis was reported in the Results and Discussion section.

### 3.4. Computational Residue Scanning of Peptide 3_10_-HBS RB3 in Complex with Ras

The 3_10_-HBS RB3 peptide in complex with Ras (from PDB 1XD2 [48]) was used to perform a computational residue scanning (Schrödinger Inc., software release v2018-4) to perform point mutations on the peptide residues. The predicted changes in binding affinity and stability were calculated according to Equation (1) [92]:

(1) $$∆∆G_{Affinity}=\left(E_{A\cdot B}^{MUT}-E_{A}^{MUT}-E_{B}^{MUT}\right)-\left(E_{A\cdot B}^{WT}-E_{A}^{WT}-E_{B}^{WT}\right)$$

where *E* is the calculated energy of each protein (A and B) or complex (A∙B) after refinement while considering the mutant form (MUT) and the wild-type (WT) of the protein. The resulting structures were refined by selecting side-chain prediction with backbone minimization. 

For the purpose of the model, ∆∆G_stability_ was computed while representing the unfolded ligand as a tripeptide, A-X-B, where X is the residue that is mutated, and A and B are its neighbours, capped with ACE and NMA. The assumption was that the remaining interactions in the unfolded state were negligible. Thus, ∆∆G_stability_ values were calculated according to Equation (2):

(2) $$∆∆G_{Stability}=\left(E_{L\left(u\right)}^{MUT}-E_{L\left(f\right)}^{MUT}\right)-\left(E_{L\left(u\right)}^{WT}-E_{L\left(f\right)}^{WT}\right)$$

where *E*, in this case, is the calculated energy for the unfolded parent ligand (L(u)) and the folded parent ligand (L(f)) while considering the mutant form (MUT) and the wild-type (WT) of the protein [92]. The calculations were done with Prime MM-GBSA [102,103], which employs an implicit (continuum) solvation model.

### 3.5. MM-GBSA Calculations of All the Complexes Used to Perform MD

The MD outputs of Ras protein in complex with 3_10_-HBS RB3 peptide, the point-mutated peptides, and the combinatorial peptides were used to compute MM-GBSA calculations through the command line. For this purpose, the Python script “thermal_mmgbsa.py” was used. Overall, 65 MM-GBSA calculations were carried out using VSGB as a solvation model, and OLPS3 FF was set for each MD trajectory. The ΔG_binding_ values were computed for each trajectory frame according to Equation (3):

(3) $${\Delta G}_{binding}=E_{A\cdot B\;\left(minimized\right)}-E_{A\;\left(minimized\right)}-E_{B\;\left(minimized\right)}$$

where *E* is the calculated energy of complex (A∙B) or each protein (A and B) after minimization [68]. Finally, the average of ΔG_binding_ values of the entire trajectories was calculated; the results were reported above in Table 7 and Table 8 in the “Results and Discussion” section.

## 4. Conclusions

The above-described work was intended to investigate potential modifications of a patented peptide, RB3 [64], to increase its inhibitory capacity of the Ras–ERK signalling pathway involved in cocaine abuse. This peptide has been reported to work as an inhibitor targeting the interaction between Ras protein and the guanine nucleotide exchange factors [63]. In detail, assays carried out on an ex vivo model of acute striatal brain slices reported an inhibitory activity of RB3 peptide against ERK phosphorylation by significantly reducing the ERK activity, with an IC_50_ of 6 µM. The inhibitory potential of this peptide was further explored by Papale and colleagues, who highlighted that this peptide was effective in decreasing the phosphorylation of two ERK substrates, (Ser10)-acetylated (Lys14) histone H3 (pAc-H3) and S6 ribosomal protein (pS6, Ser235/236 specific site) [69,70,71], with an IC_50_ of 5.2 µM for pAc-H3 and 3.69 µM for pS6 [63]. Due to the increasing interest in the RB3 peptide, it was chosen for to improve and modify the structure, aiming at increasing its inhibitory activity to reduce cocaine relapses in drug-addicted patients. The below-described strategy allowed us to identify 18 peptides exploiting the peptide RB3 structure, including amino acid mutations, and employing an artificial construct, the hydrogen bond surrogate, to stabilise the helical conformation of the peptides. The MD simulations performed on these molecules in complex with the Ras protein registered stable and frequent contacts with key residues of the Ras protein, as known from the literature [43,61]. Furthermore, MM-GBSA calculation of the MD trajectories reported promising ΔG_binding_ average values, where, for example, the combinatorial peptide forty-three showed a ΔG_binding_ value of −123.50 kcal/mol. Interestingly, the selected combinatorial peptides showed an important interaction energy increase compared to the point-mutated ones, whereas the GB solvation term reported positive values (see Tables S3 and S5 in the Supplementary Materials). Thus, it seems that the presented combinatorial peptides’ interaction patterns were crucial for the complex stabilisation, as also observed in the MD RMSD plots (see Table S6 in the Supplementary Materials).

Therefore, the 16 selected combinatorial peptides were chosen, and the next step of this work will be the biological screening of the ERK signalling pathway by measuring the phosphorylation rate of the two ERK substrates, pAc-H3 and pS6 [69,70,71]. The results of these assays will provide crucial information about the potential of these designed peptides in inhibiting Ras activation, thus preventing molecular effects’ maladaptive behavioural manifestations associated with brain conditions in which this signalling pathway is abnormally enhanced, such as cocaine abuse and certain forms of ID and ASD.

## Figures and Tables

**Table 1:** Predicted ΔΔG values from the alanine mutations on Sos binding interface amino acids retrieved by the Robetta Computational Interface Alanine Scanning Server [87,88].

ΔΔG (kcal/mol)						
Sos aa	1XD2	1BKD	1NVW	1NVV	1NVU	1NVX
Phe929	1.54	1.48	1.64	3.00	1.53	1.47
Thr935	2.97	1.59	1.11	n.a.	3.13	3.11
Lys939	n.a.	n.a.	n.a.	4.21	n.a.	n.a.
Glu942	n.a.	n.a.	1.10	n.a.	n.a.	n.a.
Asn944	2.51	2.63	2.35	2.66	2.63	2.70

n.a. = not available.

**Table 2:** Correspondences of Sos hot-spot residues (from biological assays [61] and computational alanine scanning) to RasGRF1 amino acids identified by performing protein structures alignment.

Sos aa	Corresponding RasGRF1 aa
Phe929	Tyr1178
Thr935	Thr1184
Lys939	Phe1188
Glu942	Glu1191
Asn944	Thr1193

**Table 3:** Stable interactions established during MD trajectories of Ras-Sos and Ras-RasGRF1 complexes.

	MD on Ras-Sos Complex		MD on Ras-RasGRF1 Complex	
Ras aa	Sos aa 924–957	Interaction type	RasGRF1 aa 1173–1203	Interaction type
Tyr64	Phe929	Hydrophobic	Tyr1178	Pi–Pi stacking
	Phe930	Hydrophobic	Leu1179	Hydrophobic
			Leu1183	Hydrophobic
Tyr40	-	-	Phe1188	Hydrophobic
Gln61	Ile932	Hydrophobic	Tyr1178	1 H-bond
	Thr935	1 H-bond	Thr1184	1 H-bond
Ala59	Thr935	1 H-bond	Thr1184	1 H-bond
			Leu1183	Hydrophobic
			Val1187	Hydrophobic
			Phe1188	Hydrophobic
Ser17	Glu942	1 H-bond	Glu1191	1 H-bond
			Val1187	Hydrophobic
Ala18	Glu942	1 H-bond	Glu1191	1 H-bond
Tyr32	Asn944	2 H-bonds	Gly1192	1 H-bond
			Phe1188	Hydrophobic
Pro34	Thr940	Hydrophobic	-	-
Asp57	Lys939	1 H-bond + 1 salt bridge	-	-
Gly60	Leu934	Hydrophobic	Leu1183	Hydrophobic
Lys147	-	-	Glu1191	1 H-bond + 1 salt bridge

**Table 4:** The most stable interactions between Ras and the RB3 peptide highlighted from the MD trajectory analyses.

First MD			Second MD		
**Ras aa**	**RB3 aa**	**Interaction type**	**Ras aa**	**RB3 aa**	**Interaction type**
Tyr40	Asp1185	1 H-bond	Tyr40	Asp1185	1 H-bond
Tyr40	Phe1188	Pi–Pi stacking	Tyr40	Phe1188	Pi–Pi stacking
Tyr32	Gly1192	1 H-bond	Glu31	Gly1192	1 H-bond
Gln61	Tyr1182	1 H-bond	Arg149	Glu1198	1 H-bond + 1 salt bridge
			Gln25	Gly1192	1 H-bond

**Table 5:** Computational residue scanning results for the peptide 3_10_-HBS RB3, highlighting 16 promising mutations.

3_10_-HBS RB3 Peptide aa	Mutation	ΔΔG_affinity_ (kcal/mol)	ΔΔG_stability_ (kcal/mol)
Thr1184	Arg	−19.17	−3.67
	Met	−8.17	−3.18
Asp1185	Trp	−12.56	−7.64
	Tyr	−9.19	−3.49
	Phe	−8.26	−4.26
	Leu	−7.77	−12.61
Phe1188	Arg	−9.55	−4.12
Phe1188	His	−8.25	−8.75
Ile1189	Met	−3.59	−4.08
Glu1190	His	−3.29	−4.13
Glu1191	Ile	−6.97	−4.68
	Leu	−5.16	−3.79
	Val	−4.71	−4.08
	Thr	−4.32	−4.27
Thr1193	Arg	−3.85	−5.84
	Gln	−3.56	−5.86

**Table 6:** MM-GBSA calculation results based on MD simulations of the 16 point-mutated 3_10_-HBS RB3 peptides in complex with the Ras protein.

	First Peptide	Second Peptide	Third Peptide
**Point mutation**	T1184R	T1184M	D1185W
**ΔG_binding_ average**	−89.51 kcal/mol	−92.77 kcal/mol	−103.50 kcal/mol
**ΔG_binding_ Std. Dev.**	12.50	15.53	8.90
**ΔG_binding_ range**	−128.52 to −55.50 kcal/mol	−134.237 to −39.51 kcal/mol	−126.30 to −73.35 kcal/mol
	**Fourth Peptide**	**Fifth Peptide**	**Sixth Peptide**
**Point mutation**	D1185Y	D1185F	D1185L
**ΔG_binding_ average**	−102.50 kcal/mol	−94.84 kcal/mol	−82.07 kcal/mol
**ΔG_binding_ Std. Dev.**	22.23	8.34	9.26
**ΔG_binding_ range**	−145.44 to −40.67 kcal/mol	−120.13 to −52.39 kcal/mol	−109.19 to −32.63 kcal/mol
	**Seventh Peptide**	**Eighth Peptide**	**Ninth Peptide**
**Point mutation**	F1188R	F1188H	I1189M
**ΔG_binding_ average**	−87.49 kcal/mol	−69.58 kcal/mol	−83.12 kcal/mol
**ΔG_binding_ Std. Dev.**	11.60	15.23	10.89
**ΔG_binding_ range**	−120.32 to −57.76 kcal/mol	−111.45 to −24.94 kcal/mol	−122.63 to −44.93 kcal/mol
	**Tenth Peptide**	**Eleventh Peptide**	**Twelfth Peptide**
**Point mutation**	E1190H	E1191I	E1191L
**ΔG_binding_ average**	−73.36 kcal/mol	−78.65 kcal/mol	−95.11 kcal/mol
**ΔG_binding_ Std. Dev.**	12.64	11.67	12.89
**ΔG_binding_ range**	−110.79 to −39.82 kcal/mol	−115.33 to −43.76 kcal/mol	−140.50 to −49.67 kcal/mol
	**Thirteenth Peptide**	**Fourteenth Peptide**	**Fifteenth Peptide**
**Point mutation**	E1191V	E1191T	T1193R
**ΔG_binding_ average**	−94.42 kcal/mol	−84.50 kcal /mol	−90.18 kcal/mol
**ΔG_binding_ Std. Dev.**	10.69	12.71	10.85
**ΔG_binding_ range**	−121.24 to −58.75 kcal/mol	−116.52 to −47.48 kcal/mol	−119.87 to −59.72 kcal/mol
	**Sixteenth Peptide**		
**Point mutation**	T1193N		
**ΔG_binding_ average**	−97.15 kcal/mol		
**ΔG_binding_ Std. Dev.**	11.88		
**ΔG_binding_ range**	−127.15 to −53.43 kcal/mol		

**Table 7:** Combinatorial peptides designed based on computational residue scanning performed on the 3_10_-HBS RB3 peptide and MM-GBSA calculations on MD simulations.

Combinatorial Peptides	
YLGMYLRWLVRMELGR YLGMYLMWLVRMELGR YLGMYLRYLVRMELGR YLGMYLMYLVRMELGR YLGMYLRFLVRMELGR YLGMYLMFLVRMELGR YLGMYLRLLVRMELGR YLGMYLMLLVRMELGR YLGMYLRWLVRMEVGR YLGMYLRYLVRMEVGR YLGMYLRFLVRMEVGR YLGMYLRLLVRMEVGR YLGMYLMWLVRMEVGR YLGMYLMYLVRMEVGR YLGMYLMFLVRMEVGR YLGMYLMLLVRMEVGR YLGMYLRWLVRMETGR YLGMYLRYLVRMETGR YLGMYLRFLVRMETGR YLGMYLRLLVRMETGR YLGMYLMWLVRMETGR YLGMYLMYLVRMETGR YLGMYLMFLVRMETGR YLGMYLMLLVRMETGR	25. YLGMYLRWLVRMELGN 26. YLGMYLMWLVRMELGN 27. YLGMYLRYLVRMELGN 28. YLGMYLMYLVRMELGN 29. YLGMYLRFLVRMELGN 30. YLGMYLMFLVRMELGN 31. YLGMYLRLLVRMELGN 32. YLGMYLMLLVRMELGN 33. YLGMYLRWLVRMEVGN 34. YLGMYLRYLVRMEVGN 35. YLGMYLRFLVRMEVGN 36. YLGMYLRLLVRMEVGN 37. YLGMYLMWLVRMEVGN 38. YLGMYLMYLVRMEVGN 39. YLGMYLMFLVRMEVGN 40. YLGMYLMLLVRMEVGN 41. YLGMYLRWLVRMETGN 42. YLGMYLRYLVRMETGN 43. YLGMYLRFLVRMETGN 44. YLGMYLRLLVRMETGN 45. YLGMYLMWLVRMETGN 46. YLGMYLMYLVRMETGN 47. YLGMYLMFLVRMETGN 48. YLGMYLMLLVRMETGN

**Table 8:** MM-GBSA calculation results based on MD simulations of 3_10_-HBS combinatorial peptides not misfolded during the simulations in complex with Ras protein, and with ΔG_binding_ values lower than the reference one (−79.70 kcal/mol).

	First Peptide	Third Peptide	Eleventh Peptide
**Peptide sequence**	YLGMYLRWLVRMELGR	YLGMYLRYLVRMELGR	YLGMYLRFLVRMEVGR
**ΔG_binding_ average**	−83.46 kcal/mol	−96.80 kcal/mol	−91.39 kcal/mol
**ΔG_binding_ Std. Dev.**	9.00	9.84	11.55
**ΔG_binding_ range**	−117.54 to −55.07 kcal/mol	−123.01 to −52.38 kcal/mol	−119.67 to −61.34 kcal/mol
	**Twelfth Peptide**	**Fifteenth Peptide**	**Sixteenth Peptide**
**Peptide sequence**	YLGMYLRLLVRMEVGR	YLGMYLMFLVRMEVGR	YLGMYLMLLVRMEVGR
**ΔG_binding_ average**	−92.49 kcal/mol	−79.79 kcal/mol	−92.54 kcal/mol
**ΔG_binding_ Std. Dev.**	10.15	14.46	7.93
**ΔG_binding_ range**	−129.87 to −56.49 kcal/mol	−112.94 to −41.42 kcal/mol	−116.28 to −65.18 kcal/mol
	**Eighteenth Peptide**	**Nineteenth Peptide**	**Twentieth Peptide**
**Peptide sequence**	YLGMYLRYLVRMETGR	YLGMYLRFLVRMETGR	YLGMYLRLLVRMETGR
**ΔG_binding_ average**	−100.34 kcal/mol	−102.63 kcal/mol	−88.71 kcal/mol
**ΔG_binding_ Std. Dev.**	14.21	11.01	13.50
**ΔG_binding_ range**	−137.53 to −65.42 kcal/mol	−130.87 to −62.69 kcal/mol	−125.02 to −50.94 kcal/mol
	**Twenty-third Peptide**	**Twenty-fourth Peptide**	**Twenty-fifth Peptide**
**Peptide sequence**	YLGMYLMFLVRMETGR	YLGMYLMLLVRMETGR	YLGMYLRWLVRMELGN
**ΔG_binding_ average**	−85.53 kcal/mol	−82.31 kcal/mol	−97.24 kcal /mol
**ΔG_binding_ Std. Dev.**	11.35	10.37	14.17
**ΔG_binding_ range**	−117.73 to −42.84 kcal/mol	−116.03 to −40.57 kcal/mol	−133.33 to −60.15 kcal/mol
	**Twenty-ninth Peptide**	**Forty-second Peptide**	**Forty-third Peptide**
**Peptide sequence**	YLGMYLRFLVRMELGN	YLGMYLRYLVRMETGN	YLGMYLRFLVRMETGN
**ΔG_binding_ average**	−86.56 kcal/mol	−89.59 kcal/mol	−123.50 kcal/mol
**ΔG_binding_ Std. Dev.**	11.67	10.32	20.97
**ΔG_binding_ range**	−124.70 to −56.47 kcal/mol	−128.75 to −60.00 kcal/mol	−161.49 to −75.91 kcal/mol
	**Forty-fourth Peptide**	**Forty-fifth Peptide**	**Forty-eighth Peptide**
**Peptide sequence**	YLGMYLRLLVRMETGN	YLGMYLMWLVRMETGN	YLGMYLMLLVRMETGN
**ΔG_binding_ average**	−96.31 kcal/mol	−86.04 kcal/mol	−91.80 kcal/mol
**ΔG_binding_ Std. Dev.**	17.98	13.60	9.51
**ΔG_binding_ range**	−137.97 to −57.81 kcal/mol	−124.08 to −53.76 kcal mol	−122.78 to −60.84 kcal/mol

## Data Availability

Data sharing not applicable.

## Supplementary Materials

The following are available online at https://www.mdpi.com/article/10.3390/ph14111099/s1, Figure S1. FASTA sequence alignment between RasGRF1 interacting region from two different organisms (*Homo sapiens* and *Mus musculus*); Figure S2. RMSD plot of the MD simulation performed on the Ras-Sos complex (PDB 1XD2); Table S1. Plots of the H-bonds established between Ras and Sos during the MD simulation; Figure S3. RMSD plot of the MD simulation performed on the Ras-RasGRF1 complex; Table S2. Plots of the H-bonds established between Ras and RasGRF1 during the MD simulation; Figure S4. RMSD plots of first (a) and second (b) MD simulations performed on the Ras-RB3 peptide complex; Figure S5. RMSD plot of the MD simulation performed on the Ras protein in complex with the 3_10_-HBS RB3 peptide; Table S3. ΔGbinding average values of the interaction energies and the generalized Born solvation energy for the MD trajectories of the complexes’ Ras-point-mutated 3_10_-HBS RB3 peptides; Table S4. RMSD plots of 100 ns MD simulations performed on the Ras protein in complex with the 16 point-mutated 3_10_-HBS RB3 peptides; Table S5. ΔGbinding average values of the interaction energies and the generalized Born solvation energy for the MD trajectories of the complexes’ Ras-combinatorial peptides; Table S6. RMSD plots of 100 ns MD simulations performed on the Ras protein in complex with the selected 18 3_10_-HBS RB3 combinatorial peptides; Table S7. The bar charts of protein-ligand interactions for the 18 3_10_-HBS RB3 combinatorial peptides (left column), and the plots illustrating the frequency of interaction occurrences between the combinatorial peptides and Ras protein (right column).
