# Supplementary material for: A Rational Design of α-Helix-Shaped Peptides Employing the Hydrogen-Bond Surrogate Approach: A Modulation Strategy for Ras-RasGRF1 Interaction in Neuropsychiatric Disorders
Source: Pharmaceuticals (Basel). 2021 Oct 28;14(11):1099. doi: 10.3390/ph14111099 (PMC8623491; doi:10.3390/ph14111099)

Supplementary Materials

Title: A rational design of α-helix-shaped peptides employing the hydrogen-bond surrogate approach: a modulation strategy for Ras-RasGRF1 interaction in neuropsychiatric disorders

		1198		
Homo sapiens	1184 – P P C V P Y L G M Y L T D L	A	F I E E G T P N Y T E D G L V N – 1214	
Mus musculus	1173 – P P C V P Y L G M Y L T D L	V	F I E E G T P N Y T E D G L V N – 1203	
		1187		

Figure S1. FASTA sequence alignment between RasGRF1 interacting region from two different organisms (*homo sapiens* and *mus musculus*)

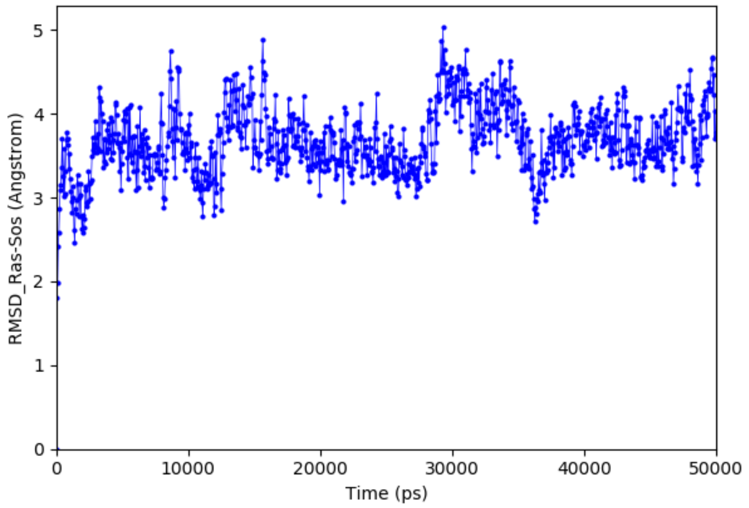

Figure S2. RMSD plot of MD simulation performed on Ras-Sos complex (PDB 1XD2)

Table S1. Plots of the H-bonds established between Ras and Sos during the MD simulation.

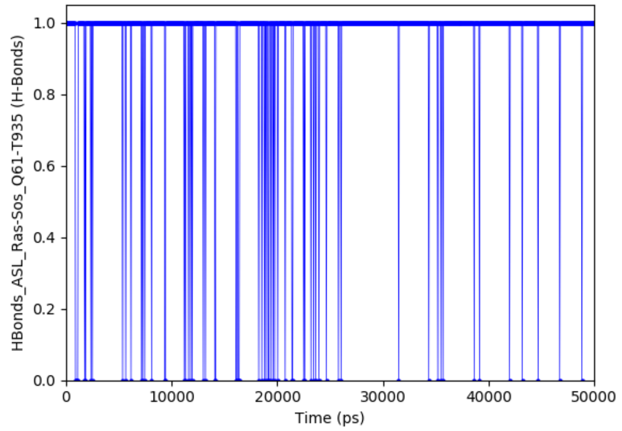 **Ras Gln61-Sos Thr935**	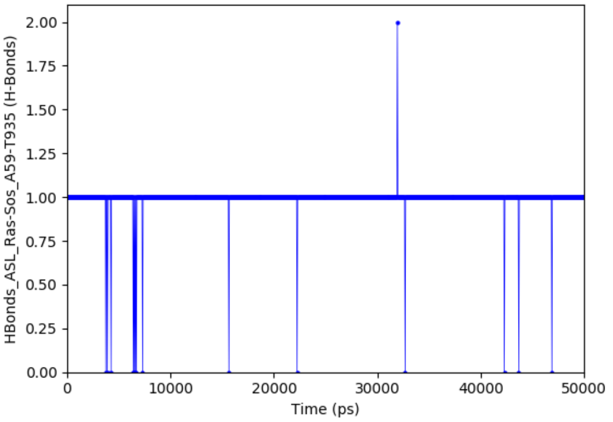 **Ras Ala59-Sos Thr935**

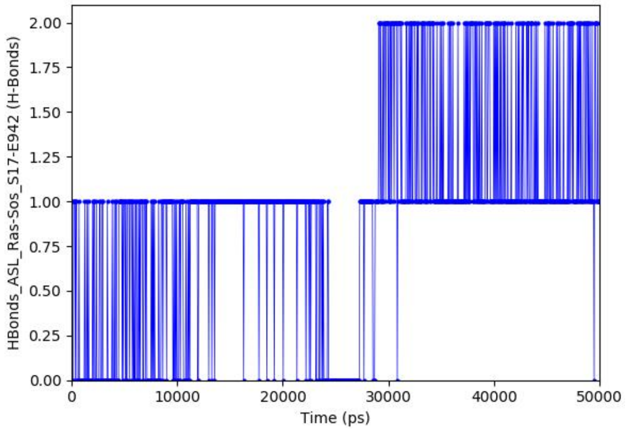

Ras Ser17-Sos Glu942

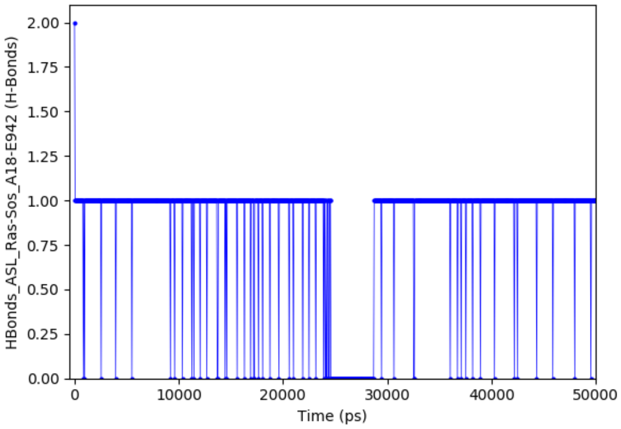

Ras Ala18-Sos Glu942

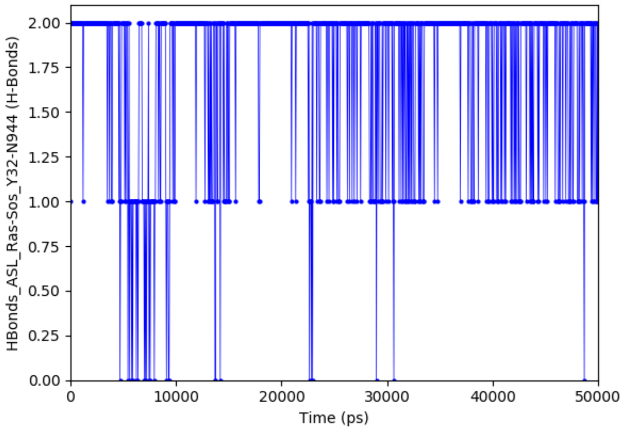

Ras Tyr32-Sos Asn944

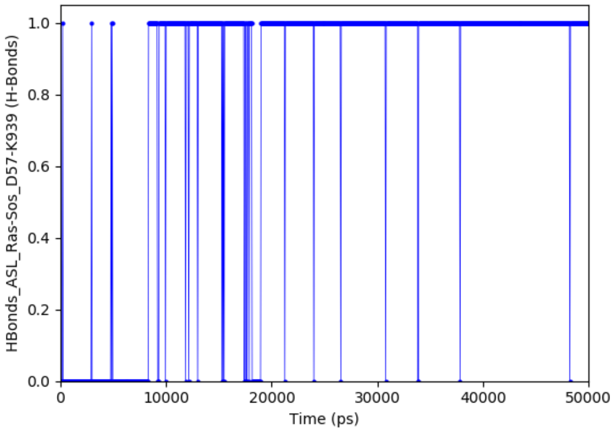

Ras Asp57-Sos Lys939

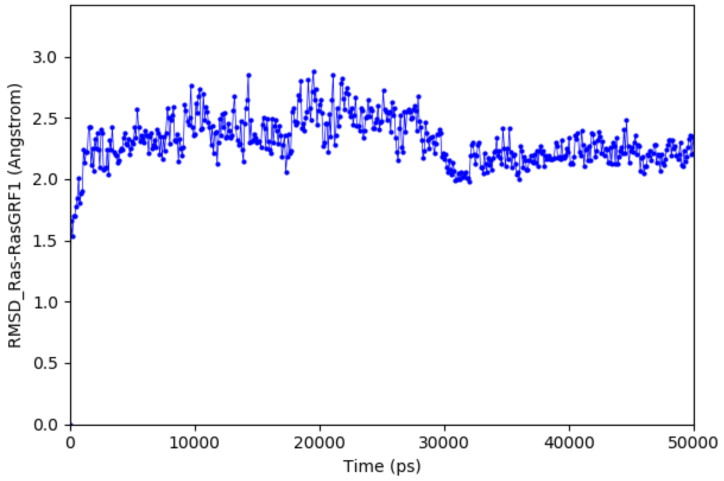

Figure S3. RMSD plot of MD simulation performed on Ras-RasGRF1 complex

Table S2. Plots of the H-bonds established between Ras and RasGRF1 during the MD simulation.

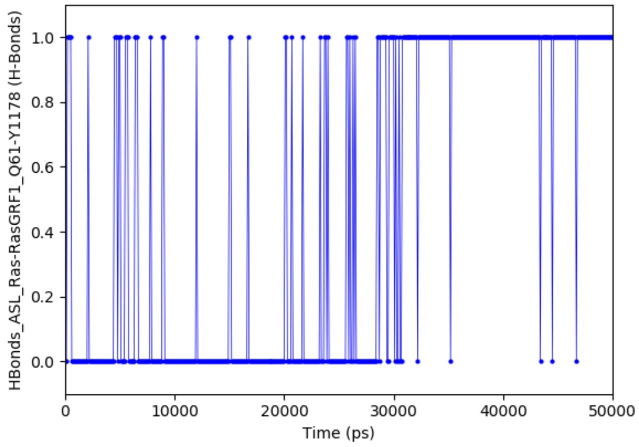

Ras Gln61-RasGRF1 Tyr1178

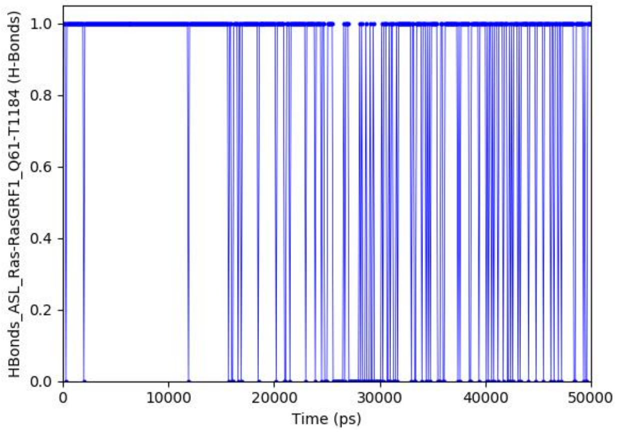

Ras Gln61-RasGRF1 Thr1184

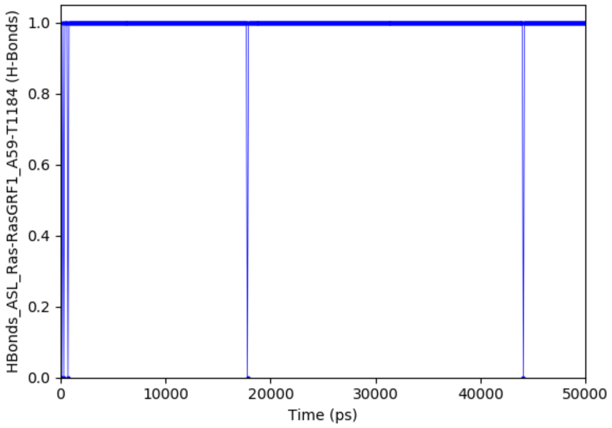

Ras Ala59-RasGRF1 Thr1184

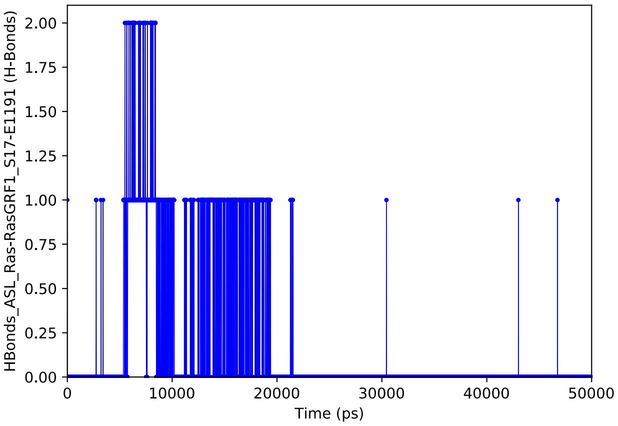

Ras Ser17-RasGRF1 Glu1191

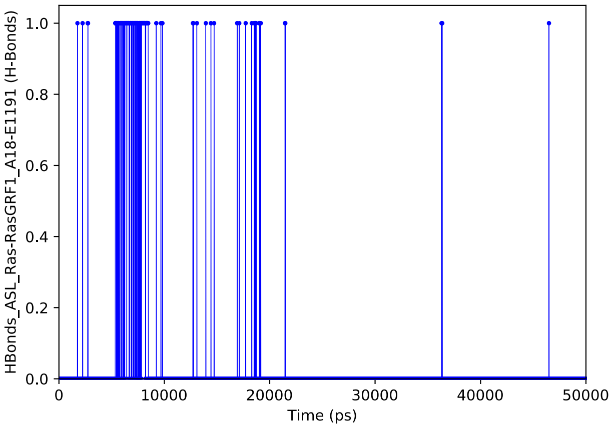

Ras Ala18-RasGRF1 Glu1191

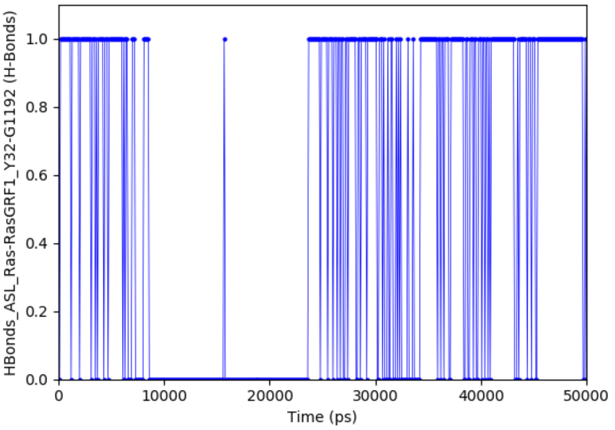

Ras Tyr32-RasGRF1 Gly1192

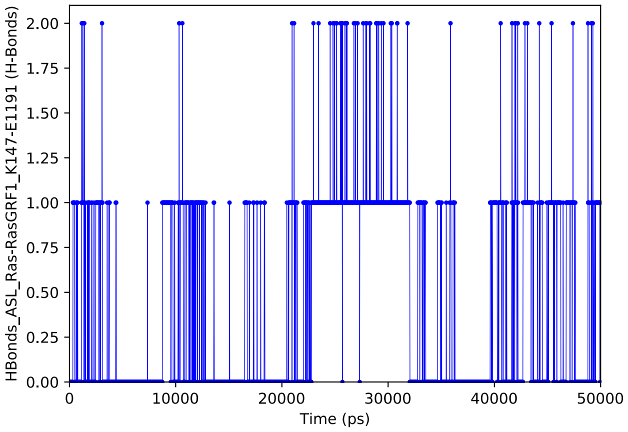

Ras Lys147-RasGRF1 Glu1191

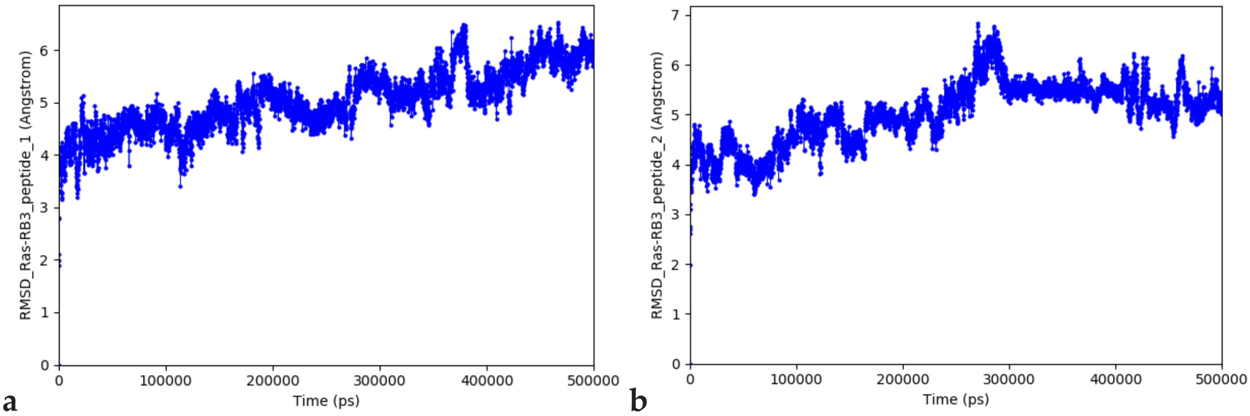

Figure S4. RMSD plots of first **(a)** and second **(b)** MD simulations performed on Ras-RB3 peptide complex

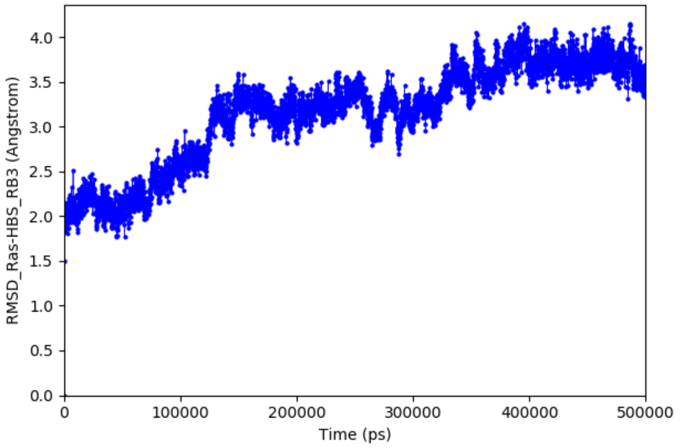

Figure S5. RMSD plot of MD simulation performed on Ras protein in complex with 3_{10}-HBS RB3 peptide

Table S3. $\Delta G_{binding}$ average values of the interaction energies and the Generalized Born solvation energy for the MD trajectories of the complexes Ras-point mutated 3_{10}-HBS RB3 peptides.

	**First Peptide**	**Second Peptide**	**Third Peptide**
**Point mutation**	T1184R	T1184M	D1185W
**$\Delta G_{binding}$ – average interaction energy**	-98.85 kcal/mol	-48.00 kcal/mol	-98.57 kcal/mol
**$\Delta G_{binding}$ – average GB solvation energy**	9.36 kcal/mol	-44.76 kcal/mol	-4.93 kcal/mol
	**Fourth Peptide**	**Fifth Peptide**	**Sixth Peptide**
**Point mutation**	D1185Y	D1185F	D1185L
**$\Delta G_{binding}$ – average interaction energy**	-101.10 kcal/mol	-84.63 kcal/mol	-78.69 kcal/mol
**$\Delta G_{binding}$ – average GB solvation energy**	-1.38 kcal/mol	-10.19 kcal/mol	-3.38 kcal/mol
	**Seventh Peptide**	**Eighth Peptide**	**Ninth Peptide**
**Point mutation**	F1188R	F1188H	I1189M
**$\Delta G_{binding}$ – average interaction energy**	-47.97 kcal/mol	-8.58 kcal/mol	-34.55 kcal/mol
**$\Delta G_{binding}$ – average GB solvation energy**	-39.52 kcal/mol	-60.99 kcal/mol	-48.57 kcal/mol
	**Tenth Peptide**	**Eleventh Peptide**	**Twelfth Peptide**
**Point mutation**	E1190H	E1191I	E1191L
**$\Delta G_{binding}$ – average interaction energy**	-36.69 kcal/mol	-44.44 kcal/mol	-53.27 kcal/mol
**$\Delta G_{binding}$ – average GB solvation energy**	-36.68 kcal/mol	-34.21 kcal/mol	-41.85 kcal/mol
	**Thirteenth Peptide**	**Fourteenth Peptide**	**Fifteenth Peptide**
**Point mutation**	E1191V	E1191T	T1193R
**$\Delta G_{binding}$– average interaction energy**	-53.11 kcal/mol	-42.78 kcal /mol	-45.44 kcal/mol
**$\Delta G_{binding}$ – average GB solvation energy**	-41.30 kcal/mol	-41.72 kcal/mol	-44.74 kcal/mol

	Sixteenth Peptide
**Point mutation**	T1193N
**$\Delta G_{binding}$ – average interaction energy**	-51.05 kcal/mol
**$\Delta G_{binding}$ – average GB solvation energy**	-46.10 kcal/mol

Table S4. RMSD plots of 100 ns-long MD simulations performed on Ras protein in complex with the sixteen point-mutated 3_{10}-HBS RB3 peptides

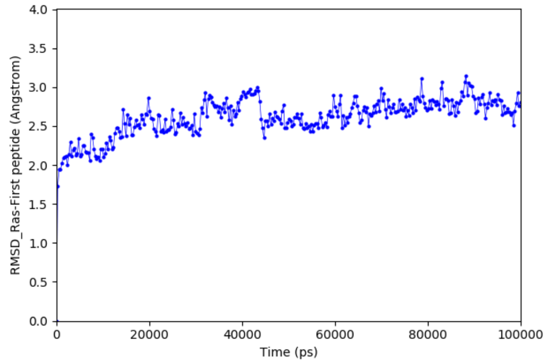

Ras-First Peptide

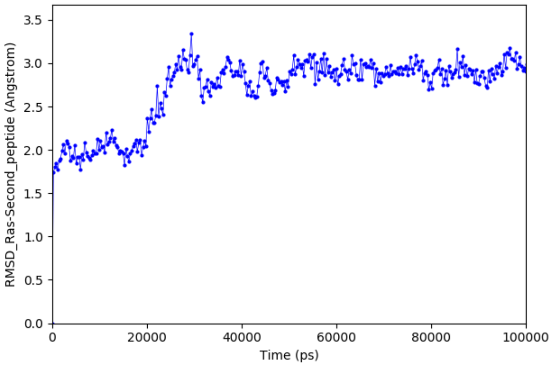

Ras-Second Peptide

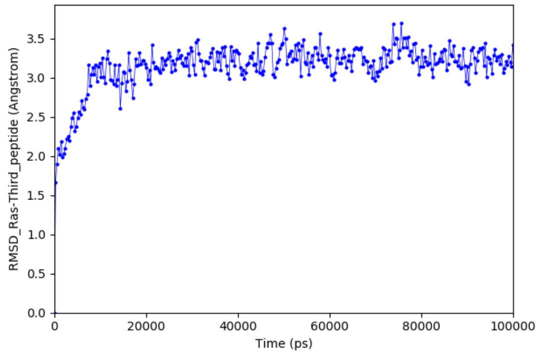

Ras-Third Peptide

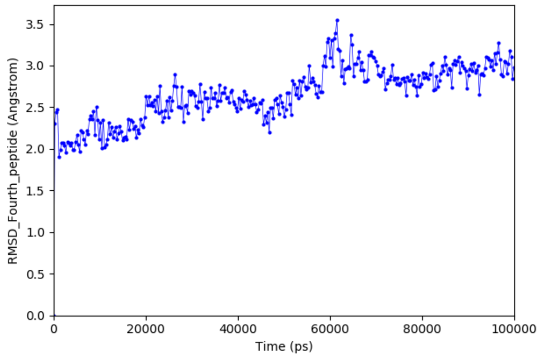

Ras-Fourth Peptide

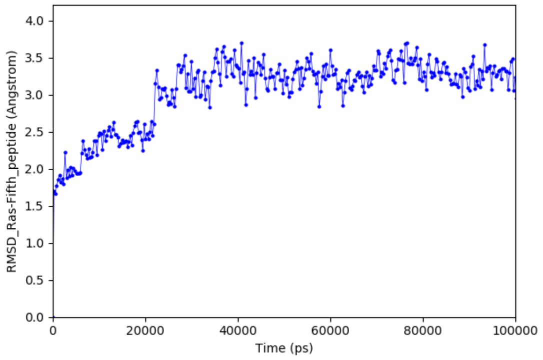

Ras-Fifth Peptide

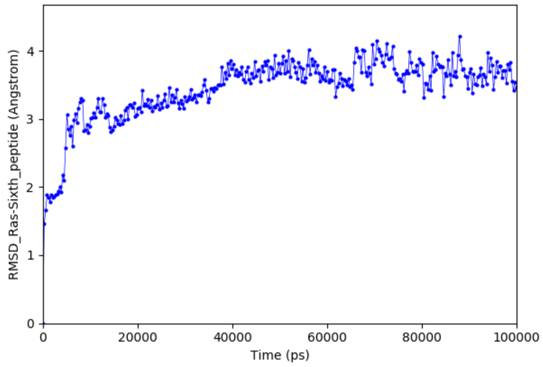

Ras-Sixth Peptide

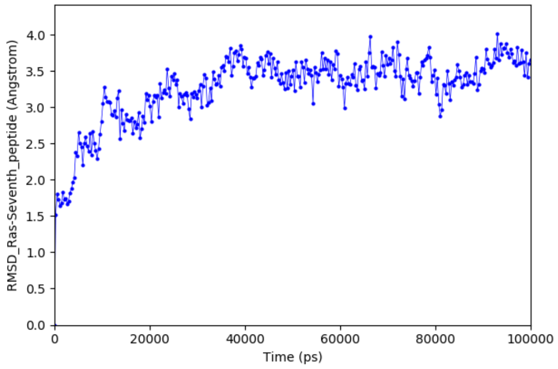

Ras-Seventh Peptide

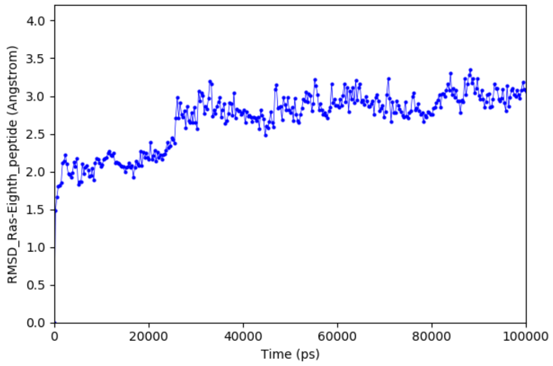

Ras-Eighth Peptide

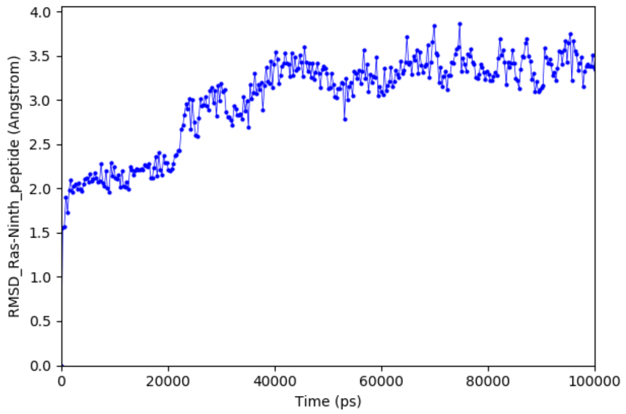

Ras-Ninth Peptide

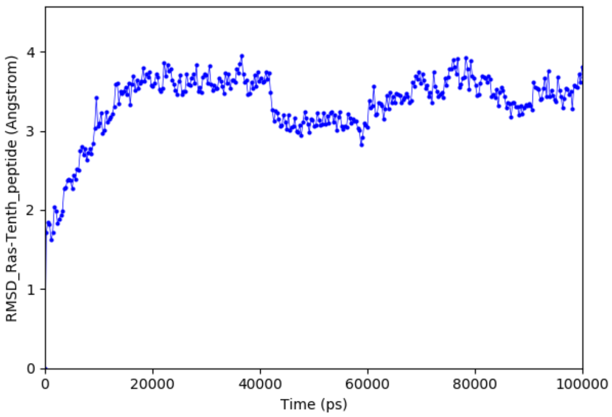

Ras-Tenth Peptide

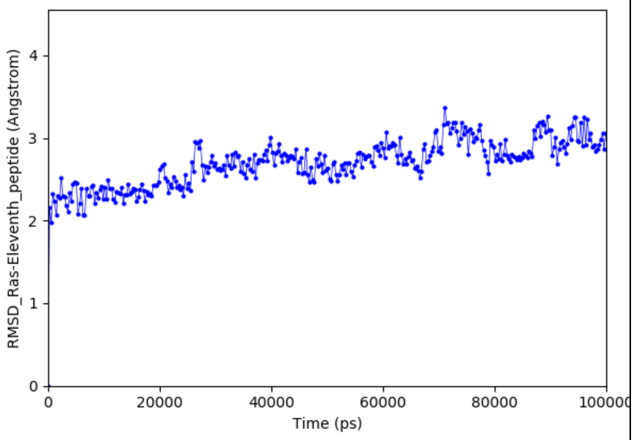

Ras-Eleventh Peptide

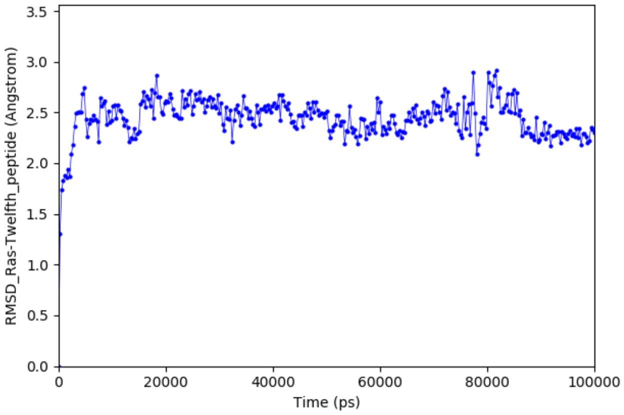

Ras-Twelfth Peptide

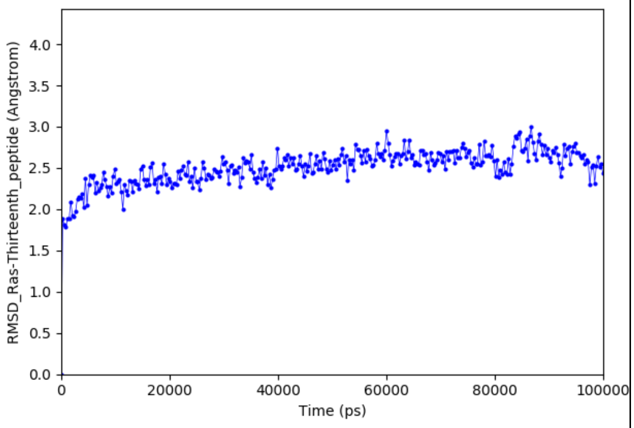

Ras-Thirteenth Peptide

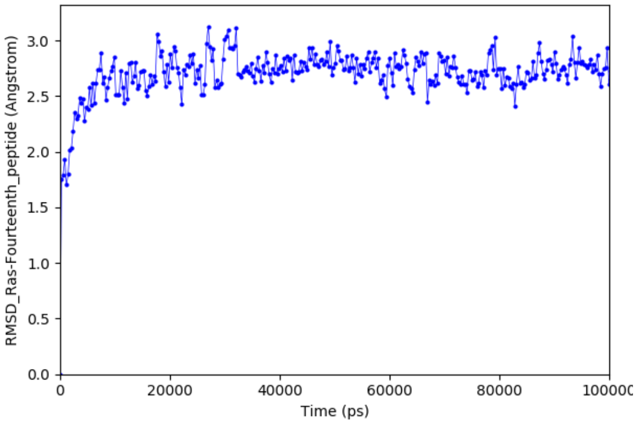

Ras-Fourteenth Peptide

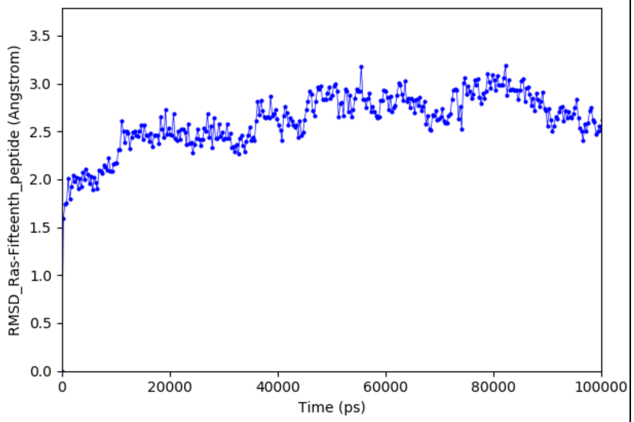

Ras-Fifteenth Peptide

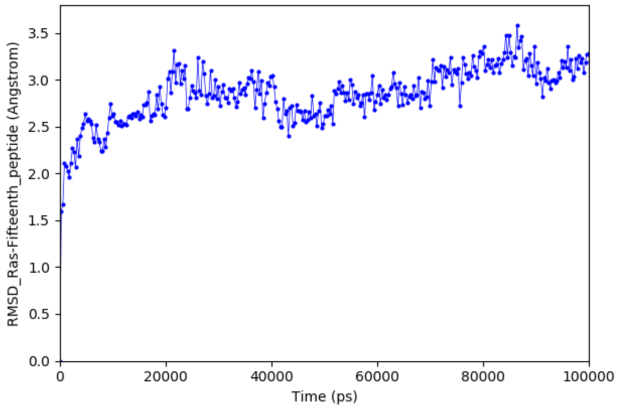

Ras-Sixteenth Peptide

Table S5. $\Delta G_{binding}$ average values of the interaction energies and the Generalized Born solvation energy for the MD trajectories of the complexes Ras-combinatorial peptides.

	**First Peptide**	**Third Peptide**	**Eleventh Peptide**
**Peptide sequence**	YLGMYLRWLVRMELGR	YLGMYLRYLVRMELGR	YLGMYLRFLVRMEVGR
**$\Delta G_{binding}$ – average interaction energy**	-217.54 kcal/mol	-251.32 kcal/mol	-233.65 kcal/mol
**$\Delta G_{binding}$ – average GB solvation energy**	134.08 kcal/mol	154.52 kcal/mol	142.26 kcal/mol
	**Twelfth Peptide**	**Fifteenth Peptide**	**Sixteenth Peptide**
**Peptide sequence**	YLGMYLRLLVRMEVGR	YLGMYLMFLVRMEVGR	YLGMYLMLLVRMEVGR
**$\Delta G_{binding}$ – average interaction energy**	-252.46 kcal/mol	-176.32 kcal/mol	-166.15 kcal/mol
**$\Delta G_{binding}$ – average GB solvation energy**	159.96 kcal/mol	96.53 kcal/mol	73.61 kcal/mol
	**Eighteenth Peptide**	**Nineteenth Peptide**	**Twentieth Peptide**
**Peptide sequence**	YLGMYLRYLVRMETGR	YLGMYLRFLVRMETGR	YLGMYLRLLVRMETGR
**$\Delta G_{binding}$ – average interaction energy**	-283.21 kcal/mol	-262.47 kcal/mol	-217.80 kcal/mol
**$\Delta G_{binding}$ – average GB solvation energy**	182.87 kcal/mol	159.85 kcal/mol	129.08 kcal/mol
	**Twenty-third Peptide**	**Twenty-fourth Peptide**	**Twenty-fifth Peptide**
**Peptide sequence**	YLGMYLMFLVRMETGR	YLGMYLMLLVRMETGR	YLGMYLRWLVRMELGN
**$\Delta G_{binding}$ – average interaction energy**	-195.79 kcal/mol	-163.02 kcal/mol	-153.99 kcal /mol
**$\Delta G_{binding}$ – average GB solvation energy**	110.27 kcal/mol	80.71 kcal/mol	56.75 kcal/mol
	**Twenty-ninth Peptide**	**Forty-second Peptide**	**Forty-third Peptide**
**Peptide sequence**	YLGMYLRFLVRMELGN	YLGMYLRYLVRMETGN	YLGMYLRFLVRMETGN
**$\Delta G_{binding}$ – average interaction energy**	-174.73 kcal/mol	-194.94 kcal/mol	-247.51 kcal/mol
**$\Delta G_{binding}$ – average GB solvation energy**	88.17 kcal/mol	105.36 kcal/mol	124.01 kcal/mol
	**Forty-fourth Peptide**	**Forty-fifth Peptide**	**Forty-eighth Peptide**
**Peptide sequence**	YLGMYLRLLVRMETGN	YLGMYLMWLVRMETGN	YLGMYLMLLVRMETGN
**$\Delta G_{binding}$ – average interaction energy**	-219.53 kcal/mol	-150.62 kcal/mol	-145.62 kcal/mol
**$\Delta G_{binding}$ – average GB solvation energy**	123.22 kcal/mol	64.59 kcal/mol	53.81 kcal/mol

Table S6. RMSD plots of 100 ns-long MD simulations performed on Ras protein in complex with the selected eighteen 3_{10}-HBS RB3 combinatorial peptides

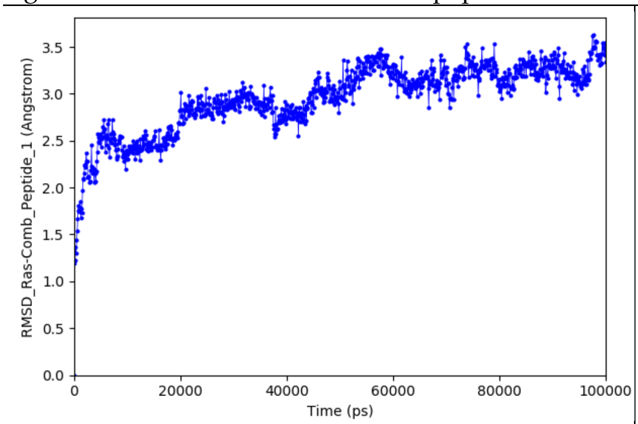

Ras-Combinatorial Peptide 1

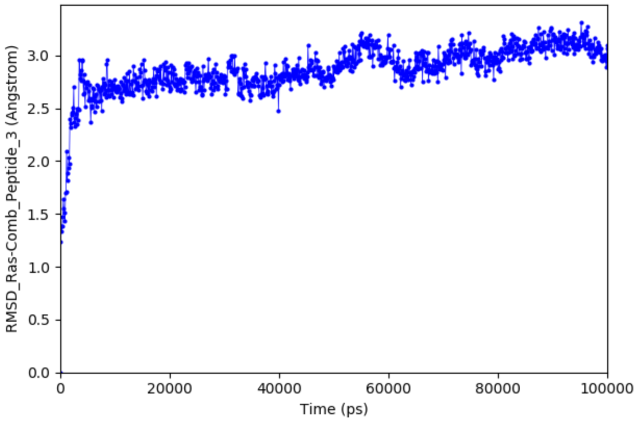

Ras-Combinatorial Peptide 3

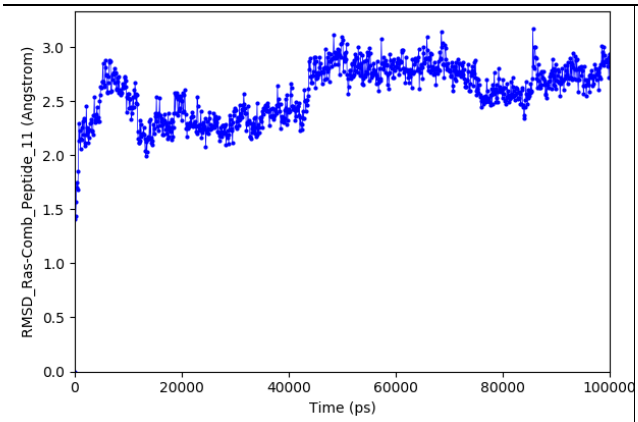 Ras-Combinatorial Peptide 11	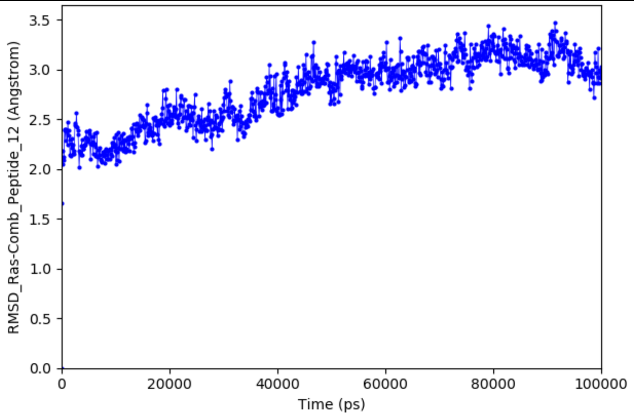 Ras-Combinatorial Peptide 12
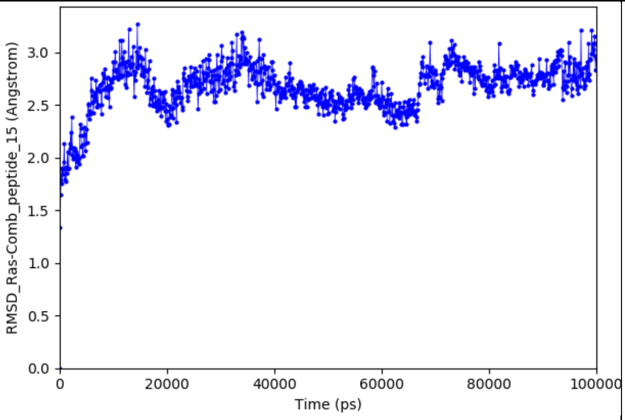 Ras-Combinatorial Peptide 15	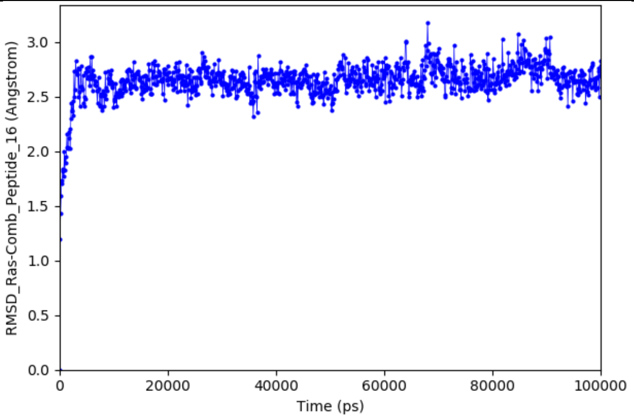 Ras-Combinatorial Peptide 16
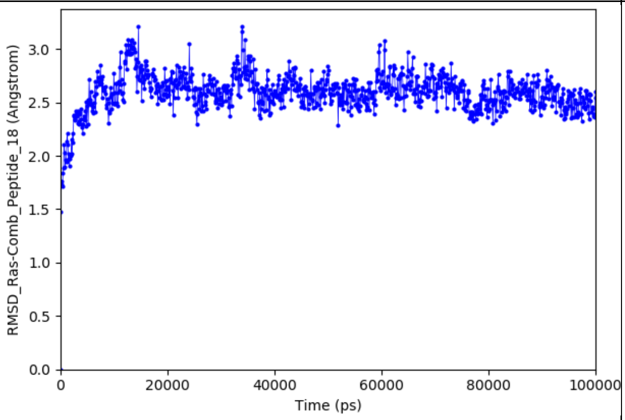 Ras-Combinatorial Peptide 18	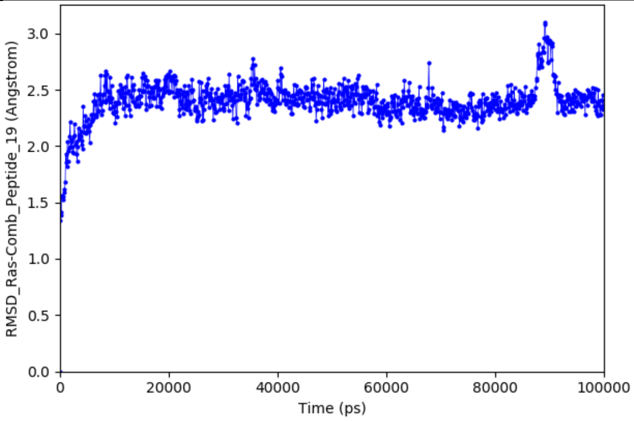 Ras-Combinatorial Peptide 19
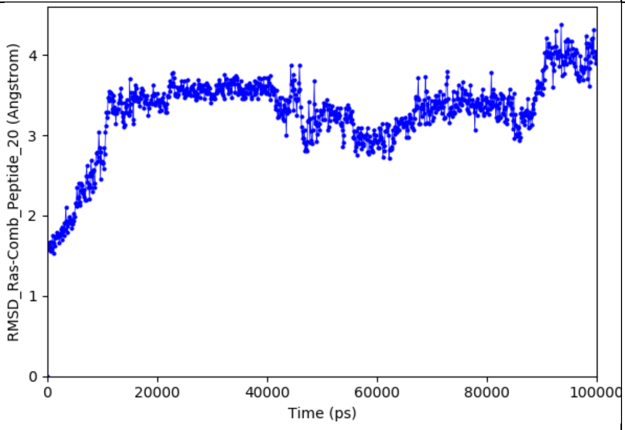 Ras-Combinatorial Peptide 20	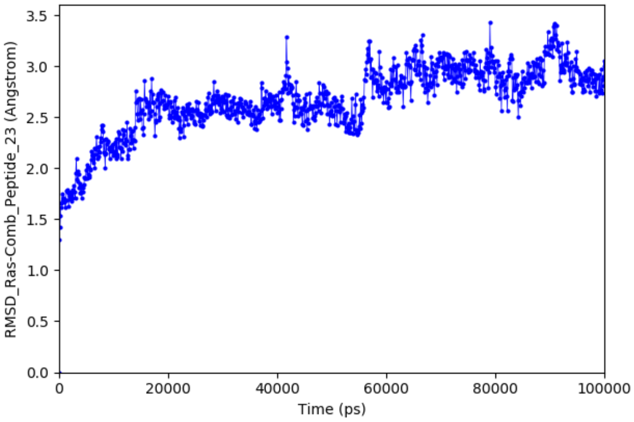 Ras-Combinatorial Peptide 23

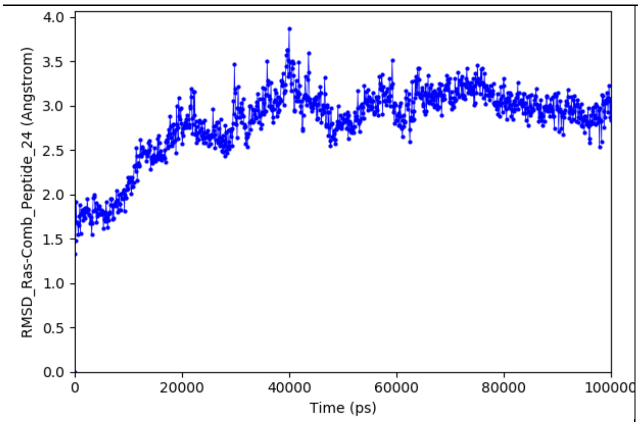

Ras-Combinatorial Peptide 24

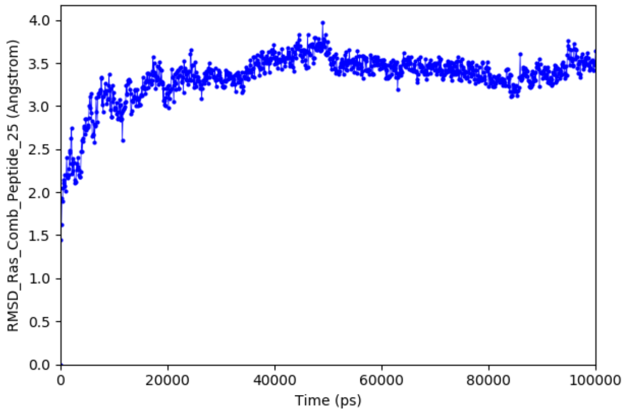

Ras-Combinatorial Peptide 25

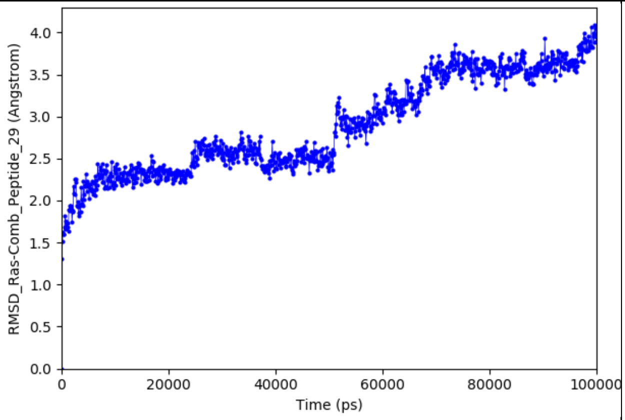

Ras-Combinatorial Peptide 29

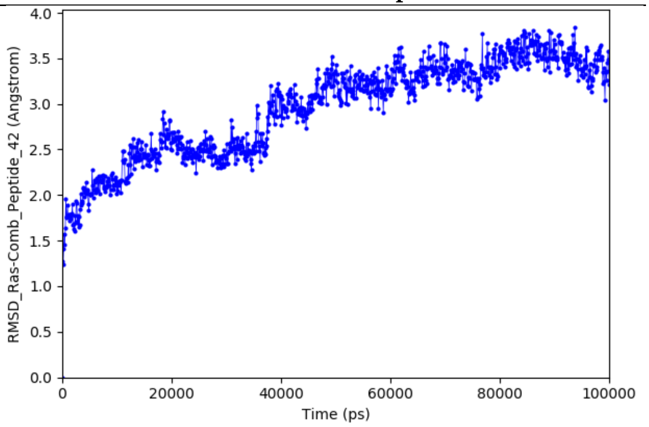

Ras-Combinatorial Peptide 42

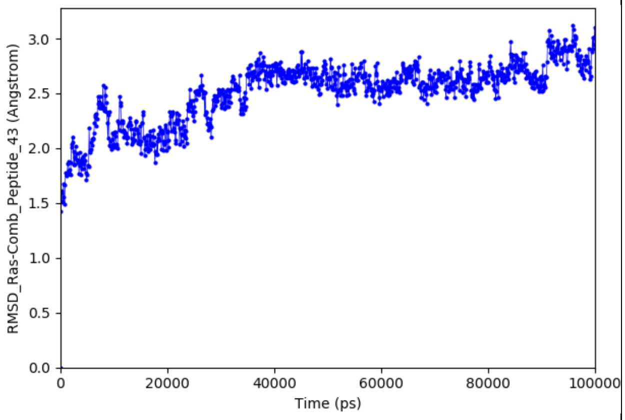

Ras-Combinatorial Peptide 43

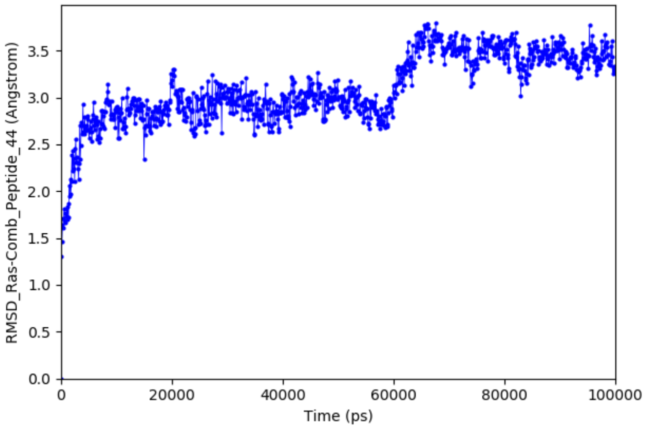

Ras-Combinatorial Peptide 44

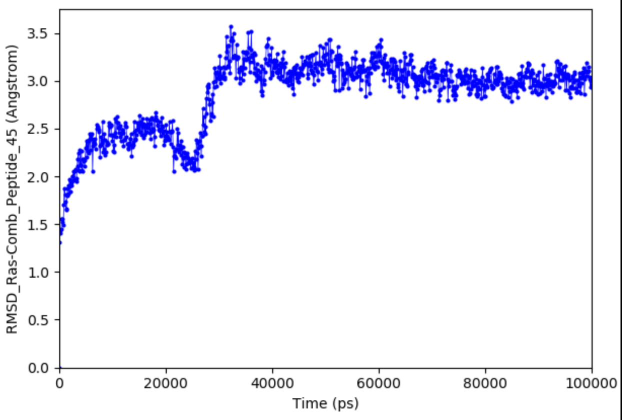

Ras-Combinatorial Peptide 45

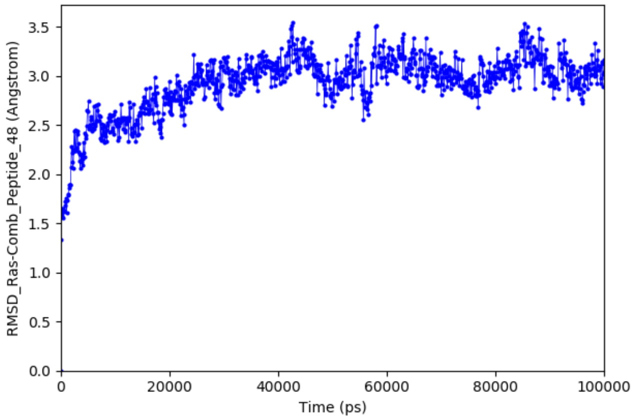

Ras-Combinatorial Peptide 48

Table S7. The bar charts of protein-ligand interactions for the eighteen 3_{10}-HBS RB3 combinatorial peptides (on the left column); the plots illustrating the frequency of interaction occurrences between the combinatorial peptides and Ras protein (on the right column)

<table>
<tr><td>Combinatorial Peptide 1</td><td>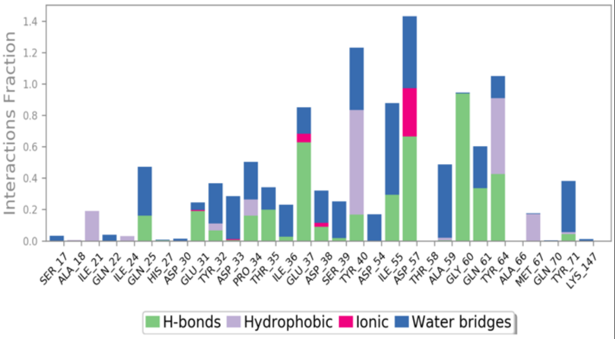
</td><td>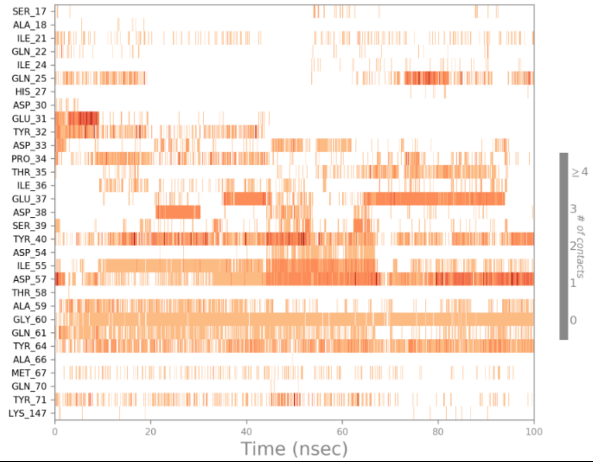
</td></tr>
<tr><td>Combinatorial Peptide 3</td><td>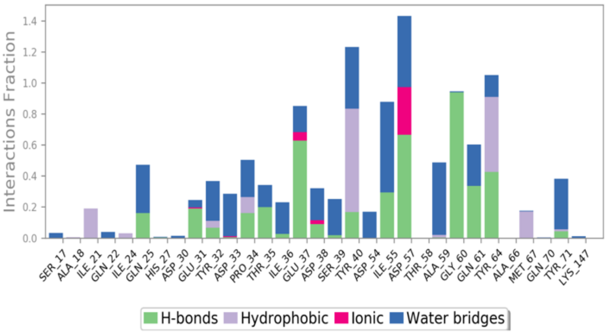
</td><td>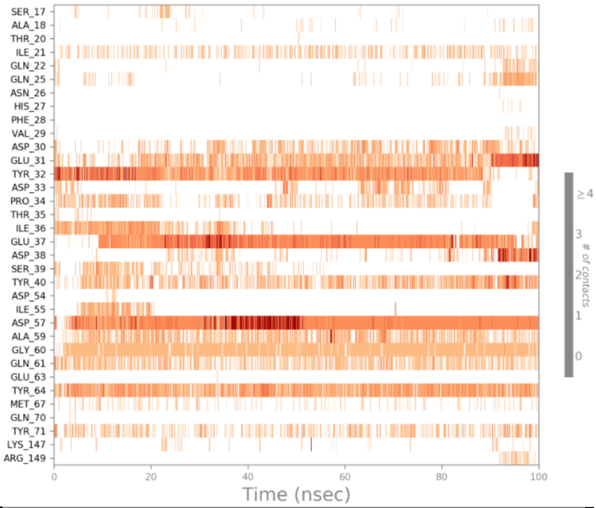
</td></tr>
<tr><td>Combinatorial Peptide 11</td><td>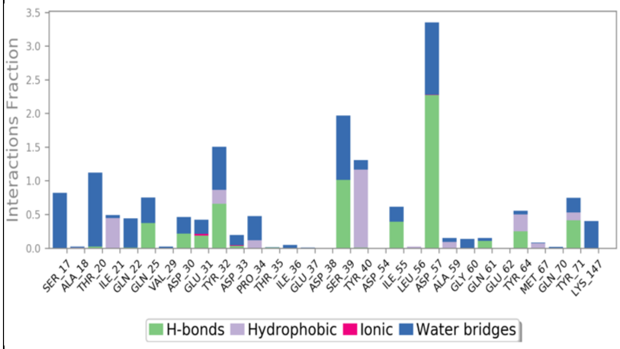
</td><td>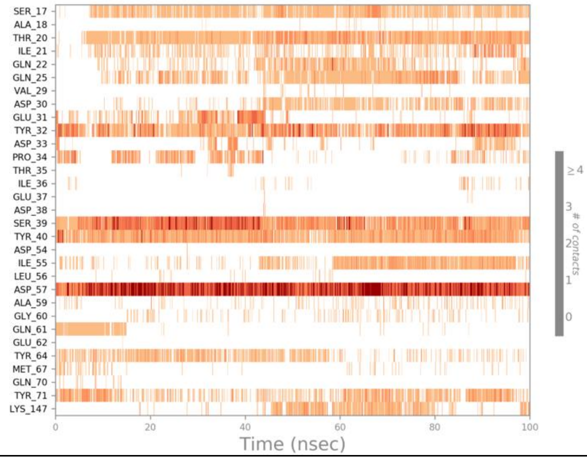
</td></tr>
</table>

Combinatorial Peptide 12

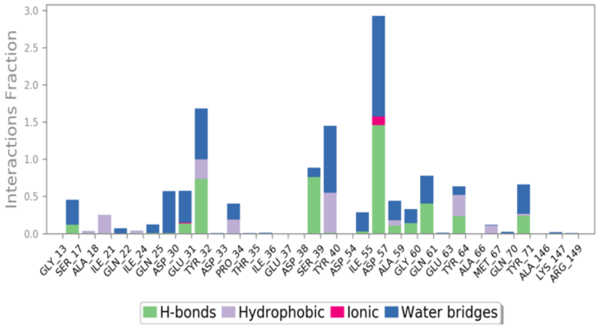

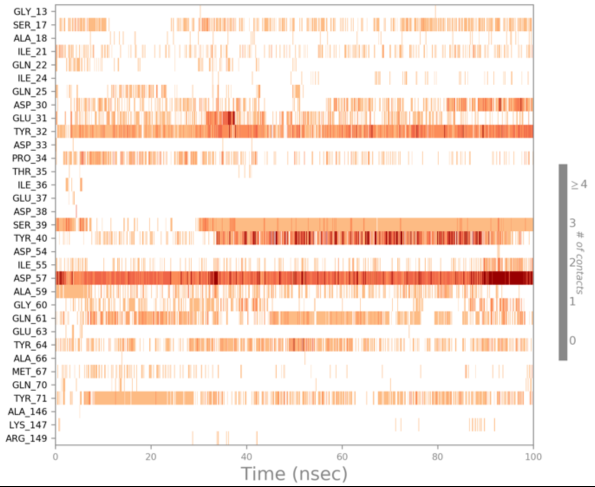

Combinatorial Peptide 15

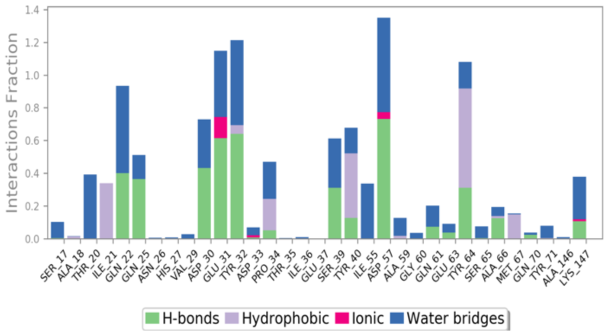

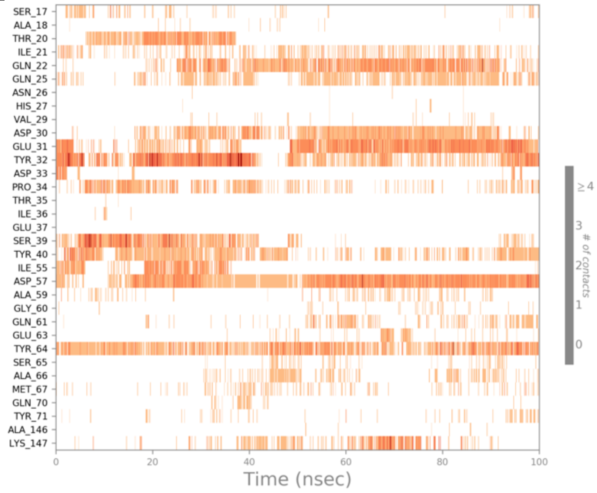

Combinatorial Peptide 16

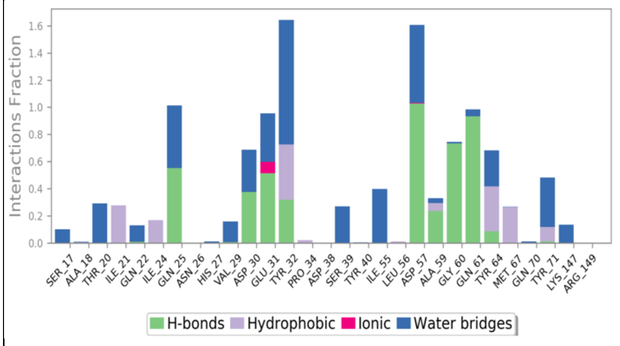

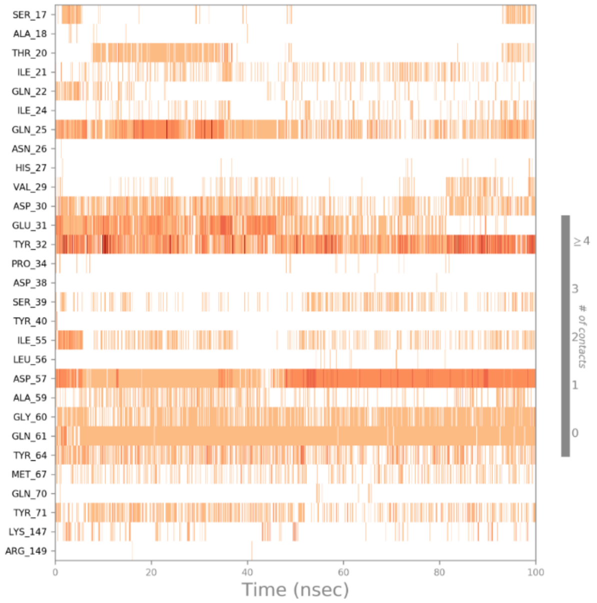

Combinatorial Peptide 18

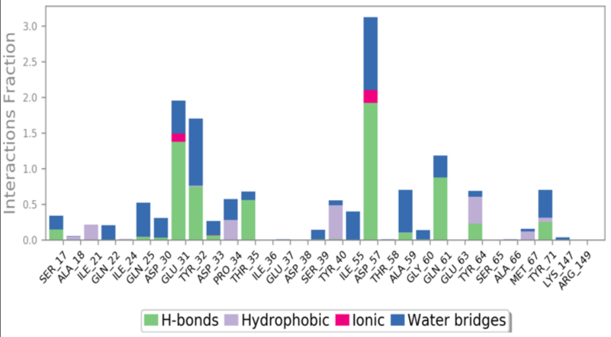

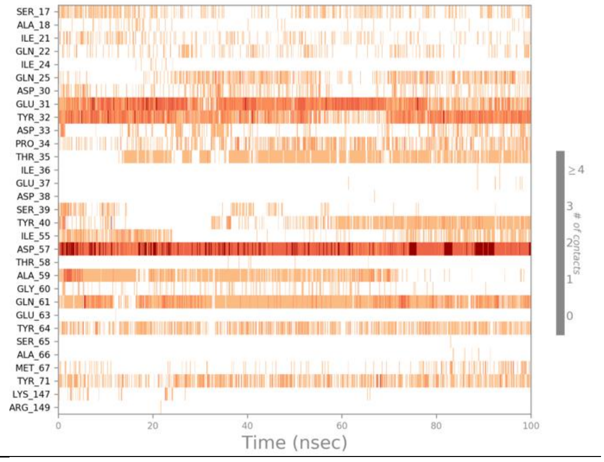

Combinatorial Peptide 19

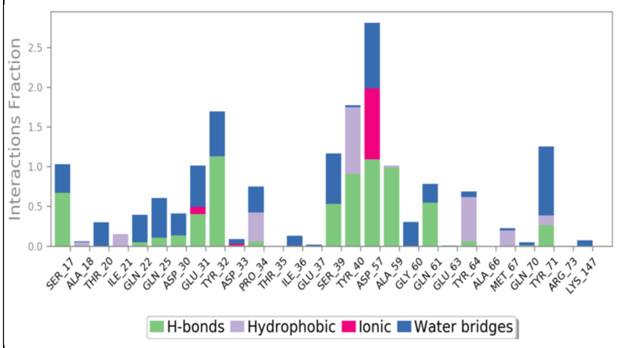

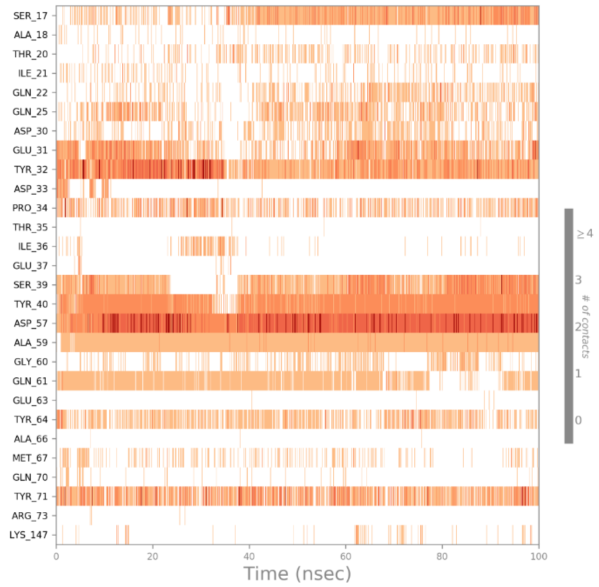

Combinatorial Peptide 20

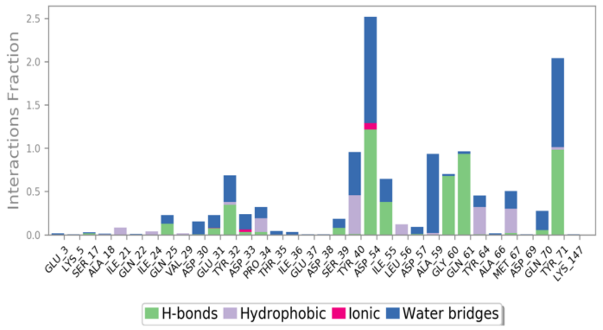

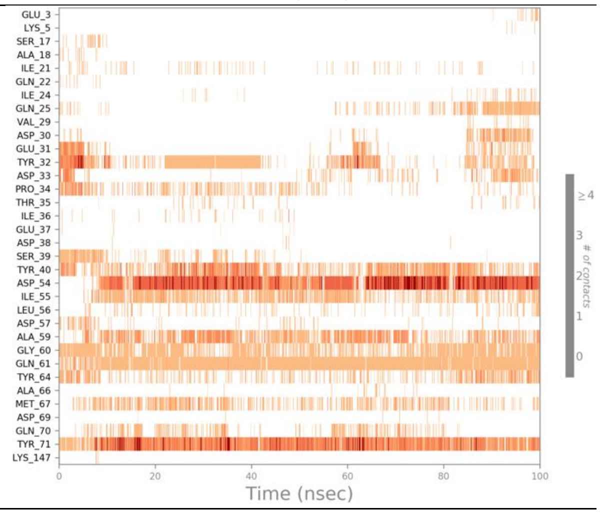

**Combinatorial Peptide 23**	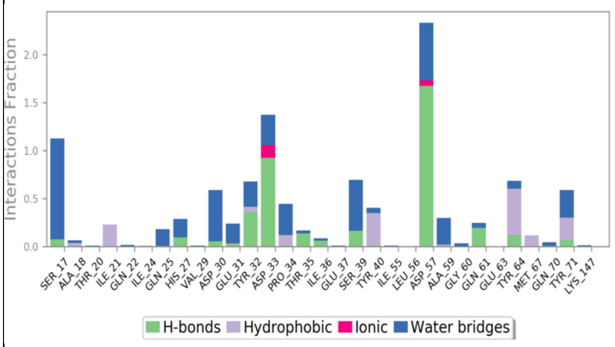	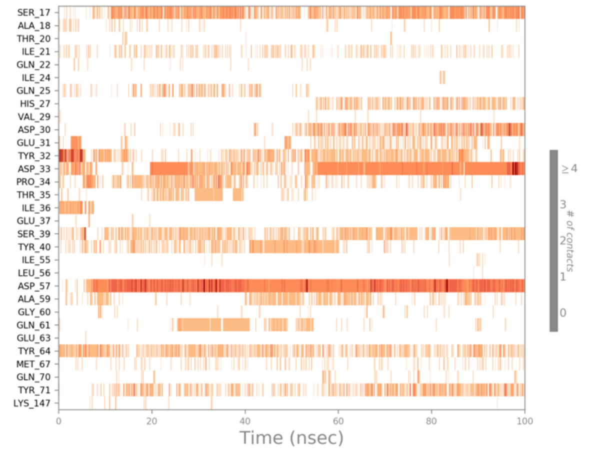
**Combinatorial Peptide 24**	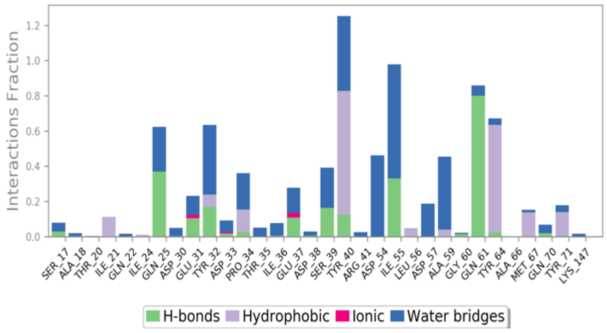	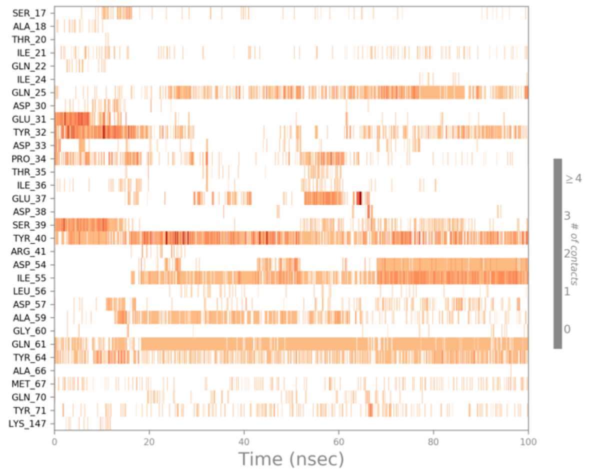
**Combinatorial Peptide 25**	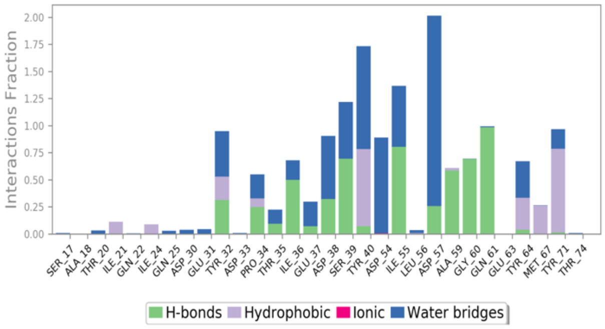	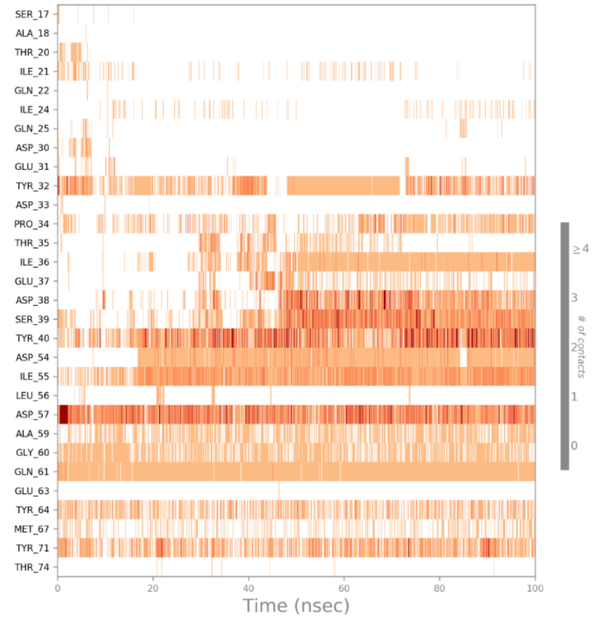

Combinatorial Peptide 29

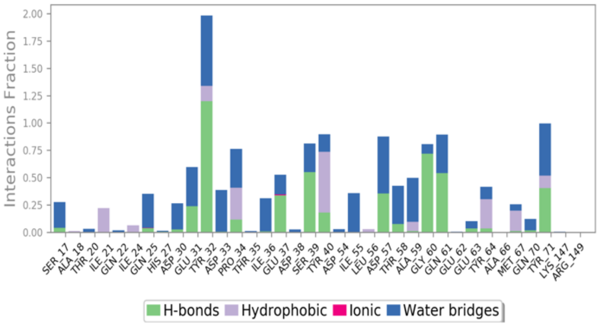

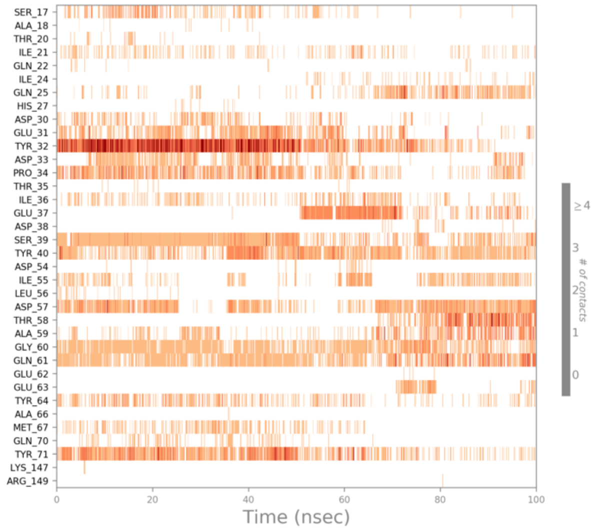

Combinatorial Peptide 42

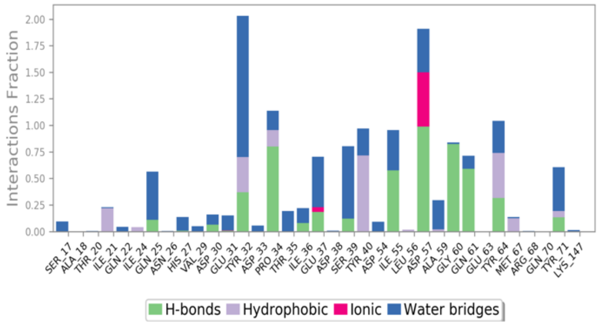

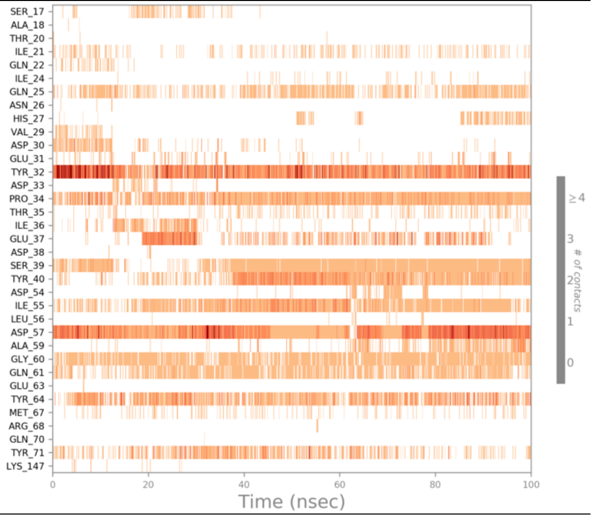

Combinatorial Peptide 43

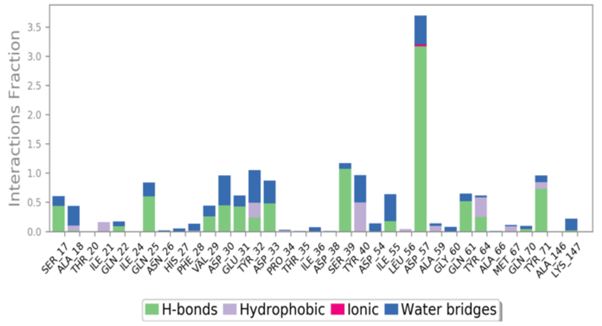

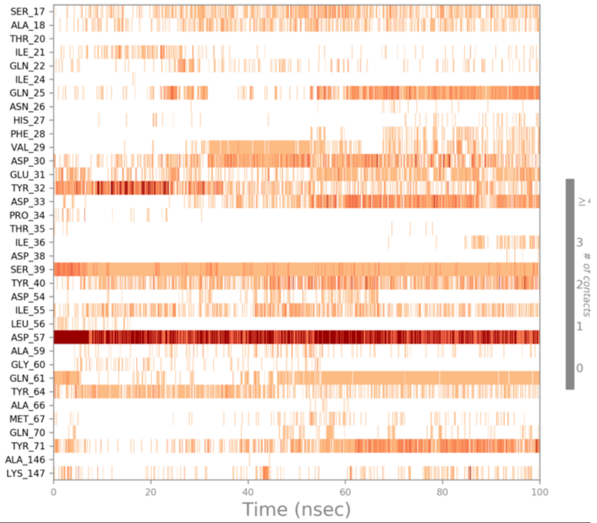

Combinatorial Peptide 44

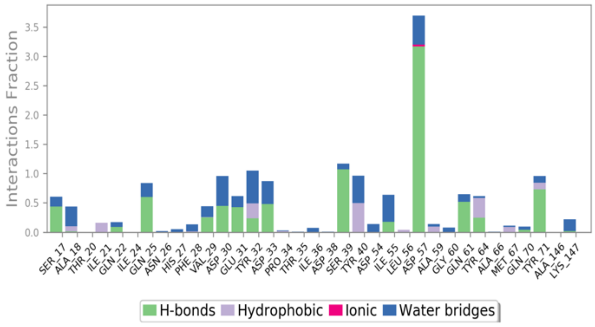

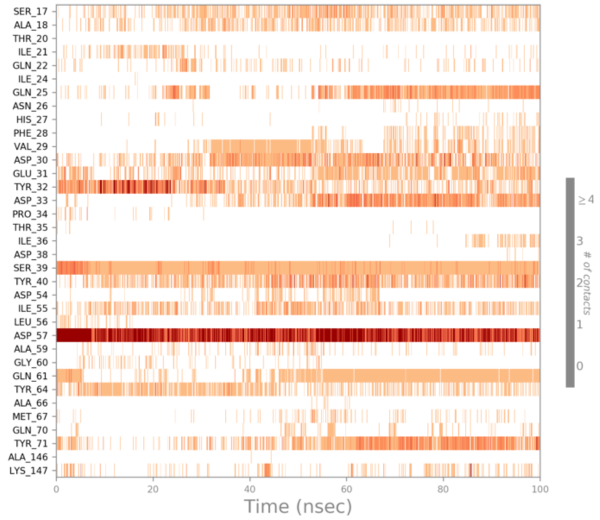

Combinatorial Peptide 45

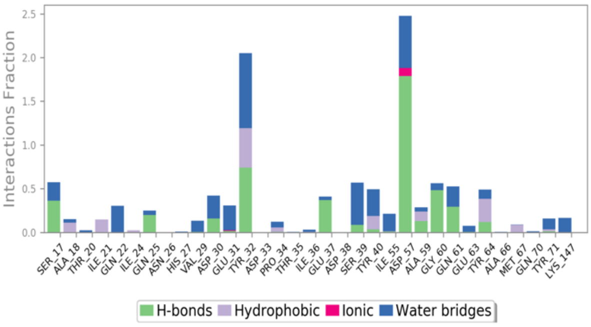

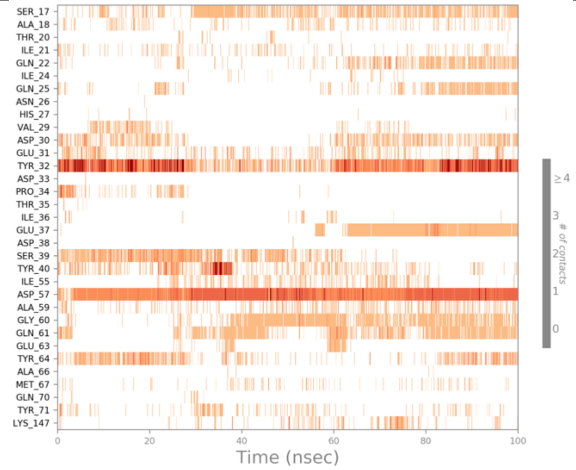

Combinatorial Peptide 48

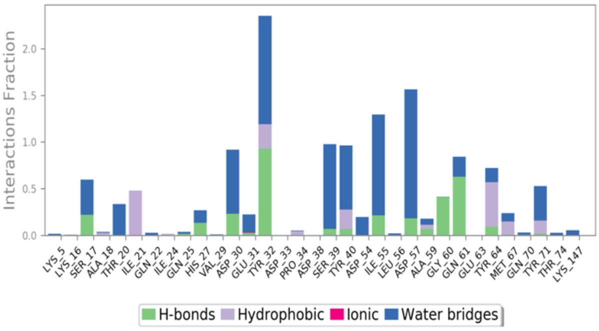

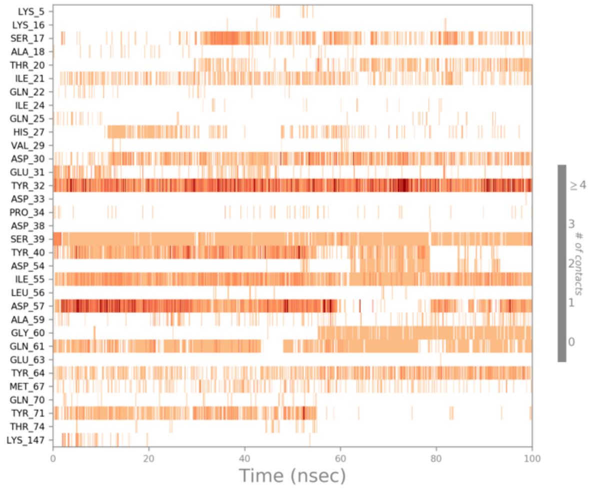

Supplement: Supplementary file 1 [file pharmaceuticals-14-01099-s001.zip › pharmaceuticals-1423595-supplementary.pdf]
